# Indigenous uses of ethnomedicinal plants among forest-dependent communities of Northern Bengal, India

**DOI:** 10.1186/s13002-018-0208-9

**Published:** 2018-01-26

**Authors:** Antony Joseph Raj, Saroj Biswakarma, Nazir A. Pala, Gopal Shukla, Vineeta, Munesh Kumar, Sumit Chakravarty, Rainer W. Bussmann

**Affiliations:** 1 Department of Forestry, Uttar Banga Krishi Vishwavidyalaya, Cooch Behar, West Bengal India; 2 Department of Forestry and Natural Resources, HNB Garhwal University, Srinagar Garhwal, Uttarakhand India; 3 Saving Knowledge, Casilla, 13092 La Paz, Bolivia; 4 Mekelle University, Mekelle, Tigray Ethiopia

**Keywords:** Tradition, Indigenous, Disease, Liver, Policy

## Abstract

**Background:**

Traditional knowledge on ethnomedicinal plant is slowly eroding. The exploration, identification and documentation on utilization of ethnobotanic resources are essential for restoration and preservation of ethnomedicinal knowledge about the plants and conservation of these species for greater interest of human society.

**Methods:**

The study was conducted at fringe areas of Chilapatta Reserve Forest in the foothills of the eastern sub-Himalayan mountain belts of West Bengal, India, from December 2014 to May 2016. Purposive sampling method was used for selection of area. From this area which is inhabited by aboriginal community of Indo-Mongoloid origin, 400 respondents including traditional medicinal practitioners were selected randomly for personal interview schedule through open-ended questionnaire. The questionnaire covered aspects like plant species used as ethnomedicines, plant parts used, procedure for dosage and therapy.

**Results:**

A total number of 140 ethnomedicinal species was documented, in which the tree species (55) dominated the lists followed by herbs (39) and shrubs (30). Among these total planted species used for ethnomedicinal purposes, 52 species were planted, 62 species growing wild or collected from the forest for use and 26 species were both wild and planted. The present study documented 61 more planted species as compared to 17 planted species documented in an ethnomedicinal study a decade ago. The documented species were used to treat 58 human diseases/ailments including nine species used to eight diseases/ailments of domestic animals. Stomach-related problems were treated by maximum number of plants (40 species) followed by cuts and wounds with 27 plant species and least with one species each for 17 diseases or ailments. Maximum number of 12 diseases/ailments was cured by *Melia azedarach* followed by *Centella asiatica* and *Rauvolfia serpentina* which were used to cure 11 diseases/ailments each.

**Conclusions:**

The list of 140 plant species indicates that the Chilapatta Reserve Forest and its fringe areas are rich in biodiversity of ethnobotanical plant species. *Rauvolfia serpentina* were the most valuable species in terms of its maximal use with higher use value. The documentation of 78 species maintained in the home gardens indicates the community consciousness on the conservation values of these ethnobotanical species. The communities should be encouraged with improved cultivation techniques of commercially viable ethnobotanical species through capacity building, timely policy intervention along with strong market linkage. This will ensure income generation and livelihood improvement and ultimate conservation of these species.

## Background

Due to globalization and green revolution, natural resources are rapidly dwindling due to the unsustainable anthropogenic activity. Consequently, the primary challenge to human society is the steady change in climate, reduction in biodiversity and dependence on external resources without giving emphasis on the enriched localised natural resources. In this setting, there is a need to explore the indigenous knowledge base for ecological, economic and environmental sustainability. Especially, forest fringe communities are associated with the forest for maintaining their livelihoods. Use of medicinal plants to treat various diseases has been part of human culture since ancient times [1]. Botanically derived medicinal plants play a major role in human society [2]. Traditional medicine forms a valuable resource for the development of new pharmaceuticals [3]. The exploration, utilization and conservation of these ethnobotanic resources are essential for restoration and preservation of traditional and indigenous knowledge [4, 5]. This acquired knowledge about the plants is very essential to be used in the near future [6]. Moreover, in developing countries now, the trend is to incorporate traditional medicines in local healthcare system and interest has increased among the researchers to explore the huge potential of ethnomedicinal knowledge for treating various diseases [7, 8].

In India and particularly in West Bengal people living in remote and rural localities are still dependent on traditional medicines for treatments of various ailments [9–12]. Indigenous people of Indo-Mongoloid origin inhabiting Chilapatta Reserve Forest in northern part of West Bengal are still using forest-originated products for their healthcare needs due to lack of availability of modern medical facilities and poor socio-economic condition [10]. The tendency of disinterestedness in old traditions is feared by elders as a major cause of losing this wealth of knowledge in coming time soon. Since traditional knowledge on ethnomedicinal plant is being eroded through acculturation and the loss of plant biodiversity along with indigenous people and their cultural background, hence, promoting research on these plants is crucial in order to safeguard this information for future societies for sustainable use and their conservation [13, 14].

Ethnomedicinal surveys provide data and information basis for conservation and sustainable utilization of local wild plants and also contribute to preserve cultural and genetic diversity. No new plant product, particularly wild, will be accepted by the urban population without proper testimony from specialists. The present study was therefore undertaken in the forest fringe area of Chilapatta Forest of West Bengal having objectives (i) document the ethnomedicinal plant species used by the community, (ii) tradition medicinal use and pattern and (iii) comparison of reported uses with different publications.

## Methods

### Study area

**Fig. 1:**
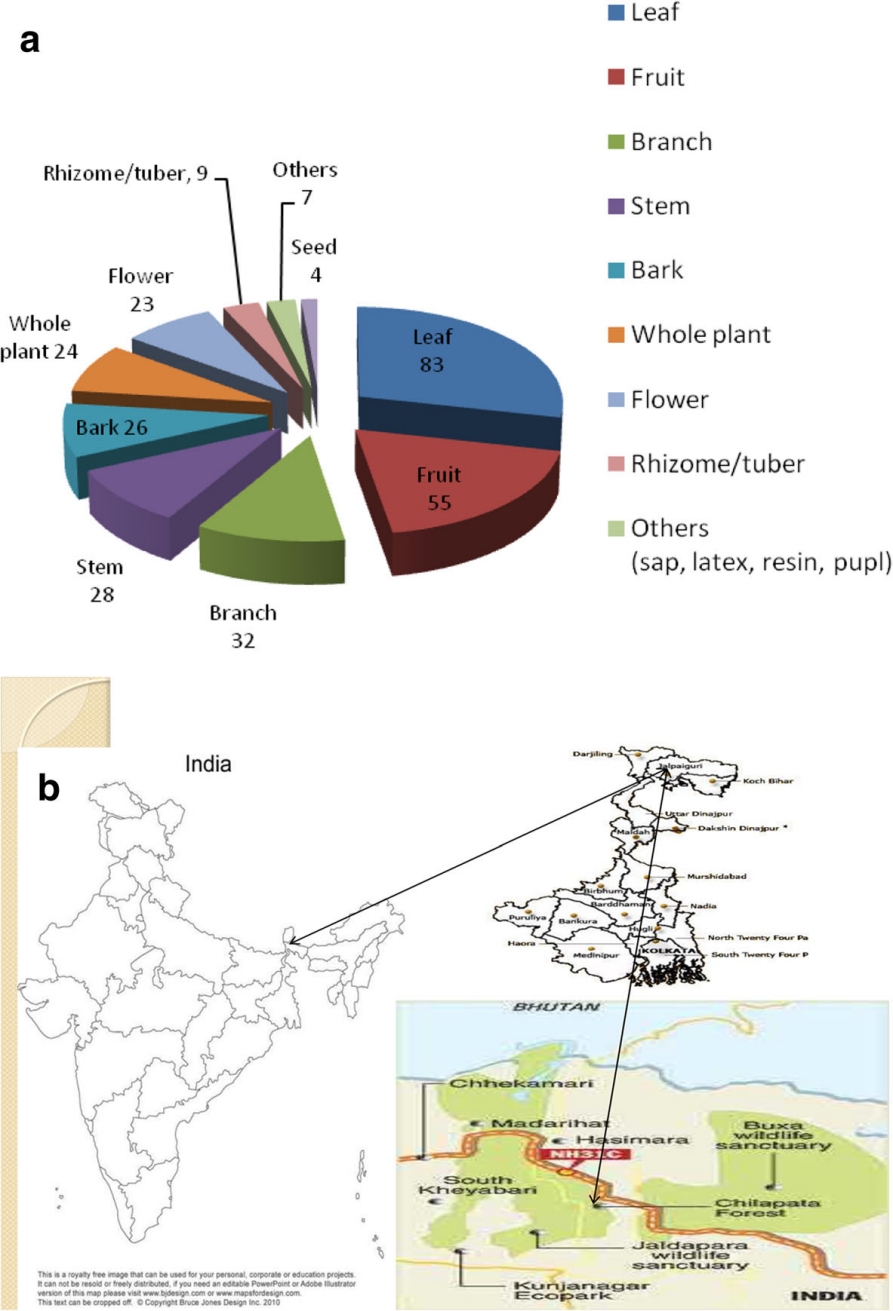
**a** Plant parts used for ethnomedicinal purposes. **b** Map of the study area

The present study was carried out at the forest fringe villages of Chilapatta Reserve Forest located in the sub-Himalayan mountain belts of West Bengal, India. The forest spreading over 41 km^2^ lies within the jurisdiction of Cooch Behar Wildlife Division in Alipurduar district (Fig. 1b). The forest is about 30 km away both from Cooch Behar and Alipurduar town, headquarters of Cooch Behar and Alipurduar district, and is transected by National Highway no. 31C. The fringe villages are Uttar Simlabari, Uttar Chaukakheti, Andu Basty, Bania Basty, Dakshin Mendabari, Uttar Mendabari, Kodal Basty, Kurmai Basty and Chilapatta Kumarpara. The elevation of the working site as measured by GPS (Garmin 72) was latitude 26° 32.85′ N and longitude 89° 22.99′ E. Mean altitude of the area was 47 m above MSL. The region is sub-tropical receiving average annual rainfall of 250–300 cm from south-west monsoon of which 80% is received from June to August. The summer and winter temperature are mild with 34 °C as the highest in the month of May while the lowest temperature is 7.5 °C during January. The forest villages with around 1000 households (average family size of 5–7 members) are inhabited by local communities of Indo-Mongoloid origin, including the Raj Bangshis, Mech, Ravas, Totos, Limbus, Lepchas, Nageshias, Uraons and Mundas. These fringe communities of the Chilapatta Reserve Forest are economically disadvantaged and thus depend on the forest and subsistence farming for their livelihoods.

### Ethnobotanical data collection

The study was conducted from December 2014 to May 2016. The villages selected were purposive. An exhaustive list of households in each village was prepared with the active cooperation of State Forest Department and local village administration (‘Panchayat’). Prior informed consent and permission to interview the villagers was obtained from the village administration and each participant verbally. A pre-tested open-ended personal interview schedule was used to elucidate aspects like plant species used as ethnomedicines, plant parts used, procedure for dosage, diseases treated and therapy. Field surveys were conducted for collecting information through interviews. Only participants over 30 years of age were considered as respondents. The age of a person was reported significantly effecting traditional knowledge [15]. Thus, a total number of 400 respondents including traditional medicinal practitioners were selected randomly. Among the respondents, 91% were males. Females did not responded to our questionnaire without their male folk; and so only those female respondents were considered who responded independently. Forty-nine per cent of the respondents were in the age range of 33–52 years, 39% in range of 53–72 years and 12% in the range of 73–92 years. Majority of the respondents (71%) have attended school up to primary level or more. The schedule was administered to the respondent in local language, and the responses were recorded in English on the schedule.

The plant specimens were collected during the survey with the help of respondents. The specimens were mounted on herbarium sheets and were identified with the herbariums of Department of Forestry, Uttar Banga Krishi Viswavidyala, Pundibari, and Department of Botany, North Bengal University, Siliguri, West Bengal. The collected information on the ethnomedicinal plants was also cross checked with published available literature. For each species, the use value (UV), as adapted by [16] from the proposal of [17], was calculated. This quantitative method evaluates the relative importance of each medicinal species based on its relative use among informants. Use value is estimated as *U*/*n*, where *U* is the number of times a species is cited and *n* is the number of informants. The use value of each species is therefore based objectively on the importance attributed by the informants and does not depend on the opinion of the researcher [16]. The collected data were analysed by using Microsoft Excel.

## Results and discussion

### Ethnomedicinal richness

A total number of 140 ethnomedicinal species represented by 116 genera and 65 families used by the indigenous communities dwelling in the fringe areas of Chilapatta Reserve Forest were documented (Table 1). Out of these, 139 species were plants and one was fungus (*Ganoderma lucidum*). Among these total species documented, 52 species were planted by the indigenous communities of forest fringe area, 62 species were growing wild or collected from the forest for use and 26 species were both wild and planted. Of these ethnomedicinally used species, trees dominated the list with 55 species (21 planted, 19 wild while 15 both growing wild and planted/domesticated) followed by herbs with 39 species (15 planted and 18 wild while 6 both growing wild and planted/domesticated), shrubs with 30 species (10 planted and 17 wild while 3 both growing wild and planted/domesticated), climber with 12 species (4 planted and 8 wild), ferns are *Christella dentata* and *Diplazium esculentum* (both wild) and least used was a creeper (*Ipomoea batatas*—planted) and a fungus (wild).

**Table 1:** List of documented ethnobotanic plants used by the communities living in fringe villages of Chilapatta Reserve Forest

1	2	3	4	5	6	7	8
Acanthaceae	*Andrographis paniculata* (Burm.f.) Wall. ex Nees	*Kalmegh, Chirawta*	0.03	H	Sp*	Pl	M, R
	*Justicia adhatoda* L.	*Basakpatta*	0.05	S	Sp*	W	M, O, Cr
	*Justicia gendarussa* Burm. f.	*Bishalyokoroni*	0.02	S		W	M, B
Acoraceae	*Acorus calamus* L.	*Ghorbaj Vasa bach, bojho*	0.02	H	Sp*	Pl	M, Cr
Amaranthaceae	*Alternanthera brasiliensis* (L.) Kuntze	*Lalful, Itingjhar*	0.03	H		Pl/W	O, N
Amaryllidaceae	*Urginea indica* (Roxb.) Kunth	*Janglipiyaz*	0.01	H		W/Pl	O
Anarcardiaceae	*Mangifera indica* L.	*Amba*	0.05	T		Pl	O, Ck
Annonaceae	*Annonas squamosa* L.	*Atafol, Saripha*	0.01	T		Pl	O
Apiaceae	*Anethum graveolens* L.	*Soya sag*	0.01	H		Pl	R
	*Centella asiatica* (L.) Urb.	*Bang Sag, Thankuni, Gortapre*	0.13	H	Co*	W	M, O, Ck, N, B
	*Centella annua* M. Schub. & B.-E. van Wyk	*Mana-muni, Beng sag*	0.04	H		W	M, O, B
Apocynaceae	*Alstonia scholaris* (L.) R. Br.	*Chatian*	0.04	T	Fr*, Sp**	W/Pl	M, O
	*Calotropis procera* (Aiton) R. Br.	*Akhanda, Akwanpata*	0.02	S		Pl	R, B
	*Rauvolfia serpentine* (L.) Benth ex Kurz.	*Nakbail, Sarpaganda*	0.11	H	E*	Pl/W	M, R, O, Cr, N
	*Tabernaemontana divaricata* (L.) R. Br. ex Roem. & Schult.	*Baramasheful, Setoful, Tagarful*	0.02	S	R**	W	O, N
	*Thespesia populnea* (L.) Sol. ex Corrêa	*Kanaliful, Karabiful*	0.01	S		Pl	Ck
Araceae	*Colocasia esculenta* (L.) Schott	*Ban-kachu*	0.01	H	Sp**	Pl/W	R
Arecaceae	*Areca catechu* L.	*Supari, Goi)*	0.03	T		Pl	R, N
	*Cocos nucifera* L.	*Nariyal, Narkel*	0.02	T		Pl	R
	*Phoenix sylvestris* (L.) Roxb.	*Khejur*	0.01	T		W	O
Asclepiadaceae	*Hemidesmus indicus* (L.) R. Br.	*Anantamul*	0.01	H		W	M
Asparagaceae	*Asparagus racemosus* Willd.	*Satomuli, Satalu*	0.03	H	Pl*	Pl	M, O
Asteraceae	*Ageratina adenophora* (Spreng.) R.M. King & H. Rob.	*Banmara, German gach, Asamiapatta*	0.04	S	A*	W	M, O
	*Ageratum conyzoides* L.	*Uchanti, Bhusuripata, Elame*	0.01	H	A*, R**	W	O
	*Eupatorium odoratum*L.	*Asamia, Banmara*	0.02	S	Fr**	W	O
	*Tagetes erecta* L.	*Mainalibibod, Marigold, Gendaful, Saipatri*	0.03	H		Pl	O, Ck, N
Athyriaceae	*Diplazium esculentum* (Retz.) Sw.	*Khukri, Dhekia sag, Niguro*	0.05	F	Sp**	W	R
Bambucaceae	*Bombax ceiba* L*.*	*Semul*	0.02	T	Sp*, Sp**	Pl/W	R
Basellaceae	*Basella alba* L.	*Puin sag, Poi sag*	0.02	C		Pl	O
Bignoniaceae	*Oroxylum indicum* (L.) Kurz.	*Kanaidingi, Totola, Surimala*	0.09	T	Fr*, Sp**	Pl/W	R, O, N, B
Brassicaceae	*Brassica rugosa* (Roxb.) L.H. Bailey	*Raya sag*	0.01	H		Pl	R
Bromeliaceae	*Ananas comosus* (L.) Merr.	*Anaras, Bhuikathar*	0.02	H		Pl	M, O
Cactaceae	*Opuntia ficus-indica*Haw.	*Sidhugach, Fanimanasa*	0.02	S		Pl	O
Caricaceae	*Carica papaya* L.	*Papita, Mewa*	0.03	T		Pl	M, O
Chenopodiaceae	*Chenopodium album* L.	*Bethu sag (N)*	0.01	H		W	M
Combretaceae	*Terminalia arjuna* (Roxb. ex DC.) Wight & Arn	*Arjun*	0.09	T		Pl/W	M, R, O, N, Bi
	*Terminalia bellirica* (Gaertn.) Roxb.	*Boir, Bahera, Barra*	0.06	T	R*, Sp**	W	R, O
	*Terminalia chebula* Retz.	*Haritaki, Harra*	0.09	T	R*, Sp**	W	M, R, O, N
Convolvulaceae	*Cuscuta europaea* L.	*Kankor, Pahelolahara*	0.01	C		W	R
	*Ipomoea batatas* (L.) Lam.	*Sakarkanda, Mistialu*	0.02	Cr		Pl	O
	*Ipomoea carnea* Jacq.	*Karmigach, Thater*	0.01	H		W	O
Crassulaceae	*Bryophyllum pinnatum* (Lam.) Kurz.	*Patharkuchi*	0.01	H		Pl	B
Cucurbitaceae	*Coccinia coridifolia* (L.) Cogn.	*Kundri*	0.01	C		W	O
	*Coccinia indica* Wight & Arn.	*Kundli, Telakuchu*	0.01	C		Pl	O
	*Lagenaria siceraria* (Molina) Standl.	*Lou*	0.01	C		Pl	O
	*Luffa aegyptiaca* Mill.	*Gongra, Dhudol, Gherawla*	0.01	C		W	O
	*Momordica diocia* Roxb.	*Jangli karela, Ban karola*	0.02	C	Co*	W	R, O
Dilleniaceae	*Dillenia indica* L.	*Pachkol (chalta)*	0.07	T	Sp**	W	M, R, O
Dioscoreaceae	*Dioscorea belophylla* Voigt	*Janglialu, Ban alu, Gichikanda, Ghetuallu*	0.05	C	R**	W	R, O, Ck
	*Dioscorea bulbifera* L.	*Kowatumbil*	0.01	C		W	O
Diptrocarpaceae	*Shorea robusta* Gaertn.	*Sal*	0.09	T	Sp*, Fr**	W	R, O
Elaeocarpaceae	*Elaeocarpus ganitrus*Roxb. ex G. Don	Rudrax	0.01	T	E^1^	W	N
Euphorbiaceae	*Baccaurea ramiflora* Lour.	*Notko, Latkafal*	0.02	T		Pl/W	R
	*Baccaurea sapinda* (Roxb.) Müll. Arg.	*Kusum*	0.02	T		Pl/W	M, O
	*Codiaeum variegatum* (L.) Blume	*Pattabahargach*	0.01	S		Pl	O
	*Emblica officinalis* Gaertn.	*Amlai, Amla, Amloki*	0.03	T		Pl	M, N, Bi
	*Hevea brasiliensis* (Willd. ex A. Juss.) Müll. Arg.	*Loborgach*	0.01	T		Pl	N
	*Jatropha curcas* L.	*Arandi, Bharenda*	0.02	S	Sp*	Pl	M, Cr
	*Mallotus tetracoccus* (Roxb.) Kurz.	*Pethali*	0.01	T		W	O
	*Ricinus communis* L.	*Aranda,Varenda, Rahari*	0.03	S	Sp**	W	M, O
Fabaceae	*Acacia catechu* (L. f.) Willd.	*Khairgach*	0.01	T	Sp*	W	R
	*Acacia nilotica* (L.) Willd. ex Delile	*Kadamkapurgach, Babul*	0.01	T		W	O
	*Bauhinia malabarica* Roxb.	*Kanchan, Tanki, Karmai*	0.02	T		Pl/W	R, N
	*Cajanus cajan* (L.) Huth	*Rahar, Raheri*	0.03	S		Pl	M, Ck
	*Cassia alata* L.	*Chakora*	0.03	S		Pl/W	O
	*Cassia sophera* L.	*Choto-kalkasunda*	0.01	S		Pl/W	M
	*Hippocrepis emerus* (L.) Lassen	*Heranchi*	0.01	S		W	M
	*Mimosa pudica* L.	*Lajjabati*	0.01	H	Fr*, R**	W	R
	*Pongamia pinnata* (L.) Merr.	*Karanj*	0.01	T		Pl/W	O
	*Tamarindus indica* L.	*Tetul, Tittri*	0.01	T	Sp*	Pl	Ck
	*Entada rheedii* Spreng.	*Gila, Gilathakuri*	0.01	T	Sp*	W	M
	*Trigonella foerum* L.	*Methi*	0.01	H		Pl	O
Fagaceae	*Quercus castanopsis* H. Lev	*Guras*	0.01	S		W	M
Ganodermataceae	*Ganoderma lucidum* (Curtis) P. Karst.	*Kath mushroom*	0.02	Fn		W	O
Lamiaceae	*Clerodendrum viscosum* Vent.	*Bhauti, Dhatupatta, Ghentu, Bhatghato*	0.04	S	Sp**	W	O, Ck, N, B
	*Gmelina arborea* Roxb. ex. Sm.	*Gamar*	0.01	T	Fr*, Sp**	Pl/W	O
	*Leucas aspera* (Willd.) Link	*Kanshisa, Ghuma, Thumbai*	0.08	H		W	M, R, O
	*Ocimum sanctum* L.	*Tulsi*	0.08	H		Pl	M, R, O, B
	*Cinnamomum camphora* (L.) J. Presl	*Dalchini*	0.01	T		Pl/W	O
	*Machilus villosa* (Roxb.) Hook. f.	*Kawla*	0.01	T	Fr**	W	O
Lecythidaceae	*Careya arborea* Roxb.	*Kumbhi*	0.02	T	Fr**	Pl	R, O
Lythraceae	*Lagerstroemia speciosa* (L.) Pers.	*Jarul*	0.01	T	Fr**	Pl/W	R
	*Punica granatum* L.	*Darim, Bedana*	0.01	T		Pl	M
Malvaceae	*Abroma augusta* (L.) L.f.	*Ulatkambal*	0.03	S	Sp*	W	O, Cr
	*Hibiscus rosa sinensis* L.	*Pajatiful*	0.01	S		Pl	M
Melastomataceae	*Melastoma malabathricum* L.	*Datrangi*	0.01	S	R**	W	O
Meliaceae	*Azadirachta indica* A. Juss	*Neemgach*	0.05	T	Pl*	Pl	R, O, Ck
	*Melia azedarach* L.	*Ghora neem, Bakain, Bakaina*	0.02	T		Pl	O
Menispermaceae	*Tinospora cordifolia* (Willd.) Miers ex Hook. f. & Thom.	*Gulancha*	0.01	C	E*, Fr**	W	M
Moraceae	*Artocarpus hetrophyllus* Lam	*Kathal*	0.03	T	Pl*	Pl	R, O, Cr
	*Artocarpus lakoocha* Wall. ex Roxb.	*Ban-kathal*	0.02	T	Sp*; Fr**	Pl/W	R
	*Ficus benghalensis* L.	*Bar rukh*	0.01	T		W	N
	*Ficus carica* L.	*Dumber*	0.01	T		W	O
	*Ficus elmeri* Merr.	*Jog dumur*	0.01	T		W	R
	*Ficus geniculata* Kurz	*Futkol, Kabragach, Pakri*	0.02	T		W	R, O
	*Ficus hispida* L. f.	*Kuchuli*	0.01	T		W	O
	*Ficus racemosa* L.	*Dumur*	0.02	T		W	O, Cr
	*Morus alba L.*	*Neel tuth, Tuth*	0.06	T		Pl	M, R, O
Moringaceae	*Moringa oleifera* Lam.	*Sajana*	0.06	T	Co*	Pl	M, R, O, N
Musaceae	*Musa balbisiana* Colla	*Kola*	0.08	H		Pl	M, R, O, Ck, Cr
Myristicaceae	*Myristica longifolia* Wall. ex Blume	*Raktakamal, Rakatgach*	0.03	T	Sp**	W	O, Cr, B
Myrtaceae	*Psidium guajava* L.	*Peyara, Asputai*	0.04	T	Co*	Pl	M, R, O
	*Syzygium cumini*(L.) Skeels	*Jamura, Jam, Jamur, Jamun*	0.02	T	Fr**	Pl	R
Oleaceae	*Nyctanthes arbor-tristis* L.	*Sefaliful*	0.02	S		Pl	O
Oxalidaceae	*Averrohoa carambola* L.	*Kamringa, Charpatay*	0.01	T		Pl	R
Piperaceae	*Piper nigrum* L.	*Golmorich*	0.01	C		Pl	Bi
Plumbaginaceae	*Plumbago zeylanica* L.	*Chitwar*	0.03	S	R*	W	M, O
Poaceae	*Arundo donax* L.	*Nolkhagra*	0.01	H		W	R
	*Bambusa vulgaris* Schrad. ex J.C. Wendl.	*Bans*	0.02	H		Pl	M, Ck
	*Cynodon dactylon* (L.) Pers.	*Dubbaghass*	0.06	H	A*	W	M, O
	*Eleusine indica* (L.) Gaertn.	*Kodoghass*	0.01	H		W	O
	*Thysanolaena latifolia* (Roxb. ex Hornem.) Honda	*Berni*	0.01	H	Pl*	Pl/W	O
	*Triticum aestivum* L.	*Ghehu*	0.01	H		Pl	O
Polygonaceae	*Polygonum dichotomum* Blume.	*Biskathali*	0.01	S		W	M
	*Polygonum hydropiper* L.	*Sukurpota, Biskutli, Pirrojhar*	0.02	H		W	M, O
Rubiaceae	*Anthocephalus cadamba* (Roxb.) Miq.	*Kadam*	0.01	T	Sp*	Pl/W	M
Rutaceae	*Aegle marmelos* (L.) Corrêa	*Bael*	0.06	T	Pl*, R**	Pl	M, O, Ck
	*Citrus limon* (L.) Osbeck	*Gololebu, Nimbu*	0.03	S		Pl	M, O, N
	*Murraya koenigii* (L.) Spreng.	*Norsing, Karipatta*	0.01	T		Pl	M
Scrophulariaceae	*Scoparia dulcis* L.	*Pith berela, Mithapatta,Chinipatta*	0.02	H	Co*	W	M, O
Simaroubaceae	*Ailanthus integrifolia* Lam.	*Gokul*	0.02	T	Sp**	W/Pl	O
Solanaceae	*Datura metel* L.	*Datura*	0.04	H		W	M, O, B
	*Solanum indicum* L.	*Bithifal, Brihati, Rambegun*	0.01	S		W	M
	*Solanum khasianum* C.B. Clarke	*Bijri kata*	0.01	S	Sp**	W	O
	*Solanum melongena* L.	*Bagun*	0.01	S		Pl	M
	*Solanum nigrum* L.	*Maichung, Kakmachi*	0.01	H	A*	W	M
	*Solanum xanthocarpum* Schrad. & J.C. Wendl.	*Kantakari*	0.01	H		Pl/W	M
Sterculiaceae	*Sterculia villosa* Roxb.	*Udal*	0.01	T	Sp**	W	N
Thelypteridaceae	*Christella dentata* (Forssk.) Brownsey & Jermy	*Bis-dhekia*	0.01	F		W	O
Typhaceae	*Typha elephantina* Roxb.	*Hogla, Bhoglapatta*	0.01	H		W	O
Verbenaceae	*Lantana camara* L.	*Ban-tulsi*	0.01	S	Sp**	W	O
	*Tectona grandis* L. f.	*Segun*	0.01	T	Fr**	Pl/W	O, M, R, Ck
	*Vitex negundo* L.	*Nisinda*	0.01	S	Fr*	W	O
Vitaceae	*Cissus quadrangularis* L.	*Harjora*	0.02	H		W	M, O
	*Cissus repanda* Vahl	*Panilarang, Panilata, Panilahara*	0.01	C		W	O
Xanthorrhoeaceae	*Aloe vera* (L.) Burm. f.	*Ghewkumari, Gritokumari*	0.03	H		Pl	R, O, N
Zingiberaceae	*Alpinia malaccensis* (Burm.f.) Roscoe	*Purundigach*	0.02	S		Pl/W	R, O
	*Curcuma caesia* Roxb.	*Kala haldi, Kalohaledo*	0.03	H		W	M, R, Ck
	*Curcuma longa* L.	*Halud, Haldi*	0.04	H	Sp*	Pl	M, O
	*Zingiber zerumbet* (L.) Roscoe ex Sm.	*Jangliadha, Jangliadhua*	0.02	H		Pl/W	M, O

1: family; 2: scientific name; 3: vernacular name; 4: use value; 5: plant form, 6: conservation status; 7: growing status; 8: community using the species

*M* Mech, *R* Rava, *O* Oraon, *Ck* Chikbaraik, *Cr* Cherwa, *N* Nepali, *B* Bengali, Bi Bihari, *T* tree, *H* herb, *S* shrub, *C* climber, *Cr* creeper, *F* fern, *Fn* fungus, *W* wild, *Sp* sparse/less frequent, *Pl* planted, *A* abundant, *Fr* frequent, *Co* common, *E* endangered, *R* rare

*[54] Chhetri et al. 2005

**[53] Shukla 2010

The tree species were represented by 44 genera and 29 families, shrubs represented by 26 genera and 17 families, herbs represented by 36 genera and 25 families, climbers represented by 10 genera and seven families, ferns represented by two genera and two families and one genus and one family each represented creeper and fungus. Trees were dominated by genus Ficus with six species and family Moraceae with nine species; shrubs were dominated by genus Solanum with three species and families Apocynaceae, Fabaceae, Euphorbiaceae and Solanaceae with three species each; herbs were dominated by genera Ageratum, Centella and Curcuma with two species each and family Poaceae with five species; and climbers were dominated by genera Coccinia and Dioscorea with two species each and family Cucurbitaceae with four species (Table 1).

The cultivated ethnomedicinal plant species were grown/planted by the respondents in their home garden, and it was found during the survey that almost all the respondents were maintaining a home garden contributing to conservation of the species they were using. Similar documentation was also reported by [10]. In total, 78 ethnomedicinal plant species were documented to be maintained in the home gardens by the indigenous community residing in and around the Chilapatta Reserve Forest of West Bengal. Similar report on home gardens maintaining rich biodiversity of ethnomedicinal plants was also reported from Ethiopia [18]. There are ample of similar documentation from the plains and Himalayan region of West Bengal including Sikkim Himalayas [18–27].

A similar study from the same study area a decade ago [10] reported 79 ethnomedicinal plant species represented by 41 families and 68 genera. This means an increment of use of 61 ethnomedicinal plant species by the community. A decade ago, the community were growing only 17 species in their home garden [10] but now, it increased to 78 species (present study). This increased the entries of ethnomedicinal plant species in the list which was documented a decade ago. This may be because of plant accessibility and visibility in the cultural landscape [28] increasing accessibility to obtain useful plants. The farther the species grows from home, the less frequently it is used, but if the plants are more desirable than well-known, species growing near home, it is worthwhile to domesticate these plants instead of undertaking long trips now and then. Plant accessibility and visibility in the cultural landscape [28] seem to have important factors influencing strategies for obtaining useful plants. Researchers conducting studies in different parts of the world indicate that knowledge of ethnomedicinal plants increases in proportion to their proximity to human habitations [29–31]. People usually know less about plants growing far from their homes and more about species that grow nearby. The same principle applies to use: people usually choose plants that grow in the immediate vicinity of their place of residence for ethnomedicinal use [32–34]. This explains the reason in increment in the number of planted ethnomedicinal plant species over a decade period in the study area.

### Ethnomedicinal uses

The documented species were used to treat 58 human diseases or ailments. Eight diseases of animals were also reported to be treated by some of the documented species (Table 2) of humans and domestic animals, respectively. The ethnomedicinal information documented for these species was also validated with earlier studies (Tables 2 and 3). Thirty plant species which were not reported in previous studies from the area. Stomach-related problems were documented to be treated by the maximum number of plants (40 species) followed by cuts and wounds with 27 plant species and least with one species each for 17 diseases or ailments (Table 3). It was noted that the common day-to-day problems (fever, stomach-related disorders cuts, wounds and burns) were treated with many species. It was documented that the communities were treating severe diseases like cancer, pox, ulcer, tuberculosis, typhoid, malaria, pneumonia and bronchitis. An earlier study on Rava community using 41 ethnomedicinal species was also documented [35]. Nine plant species were also used as ethnoveterinary medicines to cure diseases/ailments like tongue and mouth problem; cough, cold and worms; lactation problem; fatigue/weakness; diarrhoea; cuts and wounds; and appetiser (Tables 2 and 3). There are many ethnomedicinal studies that similarly documented the use of plant species used as ethnoveterinary medicines [1, 36–39].

**Table 2:** Ethnomedicinal uses of documented species and validated with earlier studies

Plants name/voucher no.	Uses (present study)	Therapy/procedure of use	Earlier studies
*Andrographis paniculata* (Burm.f.) Wall. ex Nees UBKV FOR 253	Diabetes, liver problems, fever, cough and cold, stomach pain, malaria	Whole plant is crushed, mixed with water and consumed twice daily—early morning in empty stomach and night post dinner for diabetes and liver problem. Whole plant is soaked over night, and the solution is consumed empty stomach to get relief from fever, cough, cold, stomach pain and malaria. The solution is also a tonic drink for health.	Malaria, fever [25]; poison bites, menstrual disorder [42]; for skin boils leucoderma [62]
*Justicia adhatoda* L. UBKV FOR 328	Cough and cold, paralysis, allergy, stop bleeding, cuts and wounds	5–6 leaves mixed with ginger is boiled half to its original amount and taken with honey for curing cough for 3 times a day. In serious case, it can be taken for 10–15 days approximately. Leaf paste is rubbed on cuts and wound. Leaves are boiled and the solution is used for taking bath for curing itching and stopping bleeding. Leaf extract is added with mustard oil; *Ocimum sanctum* extract and ginger extract and heated lightly. One teaspoon and half cup of this mixture is administered orally in empty stomach to children and adult, respectively for treating cough and cold. Young leaves of *Justicia adhatoda,* papaya and *Ocimum sanctum* are packed under banana leaf and heated on a pan. After heating, its half teaspoon extract is administered empty stomach once a day as an alternate therapy for cough.	Cough, cold, piles, leprosy, diabetes, bronchitis, asthma, sinusitis, anti-inflammatory [41, 66, 75, 76, 88]
*Justicia gendarussa* Burm. f. UBKV FOR 329	Cancer, septic, headache, cuts and wounds	Leaf extract is heated with coconut oil and placed on the affected part of the body for curing cancer, sceptics and cuts. The extract is also applied on cut and wound for healing and on forehead for relief from headache.	Rheumatism [83]
*Acorus calamus* L. UBKV FOR 252	Joint pain	Stem of *Acorus calamus* and leaves of *Artemisia dubia* are mixed and grinded to form a paste which is then applied on paining joints till it is cured.	Delayed delivery, eye and skin problem [25]; throat infection [42]; asthma, bronchitis, dysentery [69]; rhizome to remove lice of animals [70] cough, whooping cough, bronchitis [64]
*Alternanthera brasiliensis* (L.) Kuntze UBKV FOR 281	Cuts and wounds	Extract after crushing the twigs/leaves or flowers applied on cut or wounded part of the body and dressed.	–
*Urginea indica* (Roxb.) Kunth UBKV FOR 278	Tongue problem of domestic animals	Cloves are pasted and made solution with water and fed to suffering animals once for 2 days.	
*Mangifera indica* L. UBKV FOR 265	Jaundice, blood dysentery, diarrhoea	Tender leaves of *Mangifera indica*, *Syzygium cuminii*, and *Psidium guajava* are crushed sugar candy, mentha, *Piper nigrum* and *Centella asiatica*, and the extract is taken twice a day to cure diarrhoea. Bark is used for curing diarrhoea of domestic animals. Bark of *Mangifera indica*, *ambera* (*sadri lang*) and *Syzygium cuminii* are grinded and boiled with banana leaf and concentrated to one fourth of the initial volume. The concentrate is then administered orally to the patient to cure blood dysentery. One glass water solution of bark extract is consumed once a day in empty stomach early in the morning for 5–6 days to cure jaundice.	Indigestion, dysentery, cough and cold, worm, infection, hypertension, heat stroke**,** digestion [10, 25, 26]
*Annona squamosa* L. UBKV FOR 284.	Stomach disorders	Ripe fruit pulp are used for curing stomach problem.	Diabetes and wounds [25, 63]; ulcer, tumour [26]
*Anethum graveolens* L. UBKV FOR 283	Blood pressure	Leaves are used in curry to control pressure.	–
*Centella asiatica* (L.) Urb. UBKV FOR 257	Jaundice, typhoid, body pain, pneumonia, diabetes, cough, gastroenteritis, dysentery, stomach disorder, appetiser, dog bite, vegetable	- Leaves of *Centella asiatica*, bamboo and *Ocimum sanctum* with earthworm are boiled with water and sieved, and half a glass solution is taken for 3 times a day for 3 days to treat aundice and typhoid. - Leaves are eaten as vegetables which also help in removing body pain and fever. - Leaf extract in water solution and one glass of this solution is taken during early morning in empty stomach to heal wounds. - Roots are either eaten raw or cooked with potato (fried) for curing the pneumonia, jaundice and diabetes. - Besides this therapy, *Centella asiatica*, *Piper nigrum* and cardamom (2–3 nos.) are grinded and hot water solution is made which is orally administered for 3–4 days. - Seeds of *Piper nigrum*, barks of *Cinnamomom camphora*, roots of *halufan*, and seeds of cardamom are mixed, grinded with *Centella asiatica*, made into tablets and sun dried which is consumed with hot water three times for phenomena, joint pain, appetite and cough. - The plant/leaf extract is consumed empty stomach (2 spoons for 2–3 days) to cure gasteroenteritis, dysentery and other stomach problems. It is also consumed as an appetiser and digestive tonic. - The plant extract is mixed with sugar candy or palm candy taken 1 glass/day in empty stomach for 2–3 days to treat stomach disorders.	Constipation, indigestion, diarrhoea, dysentery, stomach problems, stomach ache, skin disease, blood related problems, diabetes, tonsillitis, cold, health and memory tonics, insomnia, blood pressure, chicken pox, stomach worm, leucorrhoea, poor urination, jaundice [10, 11, 22, 25, 35, 41, 42, 49, 55, 63, 72–74, 77, 81, 82, 87, 88, 91]
*Centella annua* M. Schub. & B.-E. van Wyk UBKV FOR 301	Gastroenteritis, stomach disorder, stomach pain, dysentery, liver problem,	The plant is either cooked as vegetable or its extract is consumed to get relief from gasteroenteritis, dysentery and stomach disorders. Its extract solution with water is taken daily as liver tonic.	–
*Alstonia scholaris* (L.) R. Br. UBKV FOR 09	Cough and cold, stomach worms of human and domestic animals, cough of domestic animals (pig), alcoholic beverage	Smelling of flowers cure cough and cold.	Increase lactation [42];
*Calotropis procera* R. Br. UBKV FOR 297	Swelling body, hydrocoel	Leaves are burnt lightly on fire and dressed on swelling area to get relief from pain. Leaves are smeared with vegetable oil and heated, then applied on scrotum to cure hydrosol. The process is continued for a week.	Nocturnal eneuresis, tumour, leprosy, dropsy, cut and wound, [25, 66]
*Rauvolfia serpentine* (L.) Benth ex Kurz. UBKV FOR 271	Paralysis, diabetes, fever, cuts and wounds, pneumonia, jaundice, stomach worm, dysentery, snake bite, gastroenteritis, stomach pain, snake repellent	Roots are eaten in empty stomach or its powder/extracts can also be taken for at least 3 months to cure diabetes. Leaves are rubbed on the body for curing paralysis. Bark is grinded adding water and kept for half an hour. This solution is taken as medicine for 3 times for curing fever and wounds. Leaf or root paste is applied on snale bite. It can simply be chewed to cure stomach pain, snake bites and dysentery. Entire plant or any other part is dried and powdered and taken in minute amount with water to cure jaundice. It should be taken in empty stomach early in the morning for 2–3 days. Root extract solution (1–2 drops for 2 days) is administered orally to cure fever or stomach worms.	Hypertension, insanity, blood pressure, fever, malaria, snake bite [10, 25, 35, 41, 62]
*Tabernaemontana divaricata* R. Br. ex Roem. &Schult UBKV FOR 14	Conjunctivitis	Flowers are simply rubbed on hands and its extract in the liquid form (only 1 drop for 2–3 days) is used to cure eye infection.	
*Thespesia populnea* (L.) Sol. ex Corrêa UBKV FOR 354	Toothache, healing, cuts andwound	–	–
*Colocasia esculenta* (L.) Schott UBKV FOR 22	Malaria, blood purifier	Fruits are cooked as vegetable and eaten with meal to cure malaria. This also cleanses blood.	Constipation, weakness, alopecia, blood circulation, cuts and injuries, liver problems, hair lengthening [22, 25, 48, 78, 85]
*Areca catechu* L. UBKV FOR 286	Periodic problems of women	Betel leaf and nuts are masticated to get relief from gastric problems. Young roots of *Areca catechu* and flowers of *Hibiscus rosa sinensis* are crushed together to extract juice. The juice is sieved, added with salt and lightly boiled which is then consumed twice daily by woman for 3–4 days.	–
*Cocos nucifera* L. UBKV FOR 309	Weight loss, hair vitalizer	Coconut milk is consumed to cure stomach problem, loose body fat and control hair loss.	Itching, sore in nose [89]
*Phoenix sylvestris* (L.) Roxb. UBKV FOR 341	Asthma, cough, dehydration, diarrhoea, fever, heart-related problems, pain, dental pain, tuberculosis	–	Inflammation and wounds, nervous debility [86]
*Hemidesmus indicus* (L.) Schultes UBKV FOR 360	Skin infection	–	–
*Asparagus racemosus* Willd. UBKV FOR 255	Cuts and wound, urine disorder, kidney and body swelling	Root extract is added with water, sieved and solution is consumed in empty stomach daily for 4–5 days for curing urine disorder. Roots are also pasted with rice, mixed with half a glass of water and consumed in empty stomach for a week or two to get relief from body and kudney swelling. Leaf extract is applied on wounds of domestic animals and dressed.	Health tonic, brain tonic, cough and cold, cut and wound, fever, diabetes, dysentery, abortion, stomach disorder, piles, tuberculosis, bronchitis, back pain [10, 25, 41, 55, 73, 74, 80, 82–85]
*Ageratina adenophora* (Spreng.) R.M. King & H. Rob. UBKV FOR 276	Massaging body, cuts and wounds	Barks and roots are washed and cooked in oil then sieved to remove the solid particles. The oil solution used to massage any body part during winter once a day for 2 days. Leaves are crushed in between the palms and then applied on cuts and bandaged.	Cuts and wounds, dysentery and jaundice, antifungal [11–13, 55, 71, 73, 74]
*Ageratum conyzoides* L. UBKV FOR 29	Cuts and wounds	Leaves are washed and crushed for extracting its juice and applied on cuts including leaves. It is tied with a piece of cloth, which results in healing cuts as well as stops bleeding.	Stomach ache, cuts and wounds, stop bleeding, antihelmenthic [64, 72, 75, 78]
*Eupatorium odoratum* L. UBKV FOR 30	Cuts and wound, bleeding	Leaf extract is applied on cuts to stop bleeding and healing as well.	Stop bleeding [35, 41]
*Tagetes erecta* L. UBKV FOR 352	Dental problem, mouth ulcer, cuts and wounds	Leaf extract is applied on cuts and dressed for healing. Flower extract is applied on mouth for healing ulcers. Leaves are chewed early in the morning to stop bleeding during brushing of teeth.	Blood coagulator, cuts and wounds, [41, 67, 82, 87]
*Diplazium esculentum* (Retz.) Sw. UBKV FOR 33	Stomach problem	Leaves are cooked as vegetable and eaten with rice for curing stomach problem.	Constipation [55, 73, 74]
*Bombax ceiba* L*.* UBKV FOR 35	Stomach pain, diarrhoea	Root extract administered orally twice a day to treat diarrhoea. Tender leaves are chewed raw in empty stomach twice a day for 2–3 days to get relief from stomach pain.	Headache, fracture, blood dysentery, pimples, skin eruptions, tooth ache, leprosy, gonorrhoea, diabetes, pimples, anaemia, and scorpion sting, virility [10, 25, 35, 41, 55, 63, 64, 72–74, 81, 88]
*Basella alba* L. UBKV FOR 292	Skin burns, blood pressure	The entire plant is pasted and applied on infected part once to get relief from burn. Cooked as vegetable to reduce blood pressure but is not taken by people having cut and wounds.	Tuberculosis, dysentery, constipation, intestinal disorder, vomiting [41, 55, 73, 74, 81]
*Oroxylum indicum* (L.) Benth. UBKV FOR 34	Jaundice, cuts and wounds, body pain, liver problem	Flowers and leaves are cooked and taken with meal for curing jaundice and regulate blood pressure. Bark soaked in water used for taking bath also cures jaundice. Bark powered and mixed in water for taking bath is used for curing pneumonia. Dried bark powder is applied on cuts and wounds to heal. Bark extract consumed early in the morning for 5–6 days cure jaundice and liver problem.	Jaundice, regulate blood pressure, cough, fever, pneumonia, stomach/chest/body pain, bronchial asthma, diarrhoea, dysentery, joint pain, appetiser, [10–12, 35, 41, 64]
*Brassica rugosa* (Roxb.) L.H. Bailey UBKV FOR 294	Skin eruptions, ulcers, headache	–	–
*Ananas comosus* (L.) Merr. UBKV FOR 282	Stomach worms	Two teaspoons of leaf extract are consumed in empty stomach daily for 2–3 weeks against stomach worms.	Leaf extract to kill worms; fruit juice against scurvy [41]
*Opuntia ficus-indica* Haw. UBKV FOR 340	Tuberculosis	Jelly-type branch after peeling is cooked with wild onion and ghee till it is completely dried which is then consumed after meal three times a day to cure tuberculosis.	–
*Carica papaya* L. UBKV FOR 298	Gastroenteritis, appetiser, digestive	Unripe fruits cooked and eaten to improve digestion or eaten raw to get relief from oral infection. Flowers are cooked as vegetable to maintain appetite. Both ripe or unripe fruits are eaten to cure gasteroenteritis.	Digestion, jaundice, dysentery, tooth ache, cough, indigestion, liver tonic, piles, heart problem, skin infection, fracture [11, 25, 55, 73, 74, 76, 78, 81, 82]
*Chenopodium album* L. UBKV FOR 302	Clearing stool, piles, gastroenteritis	Plant is cooked as vegetable and consumed for clearing stool, curing piles and gasteroenteritis.	Fever and influenza, constipation, intestinal worms [75, 76, 81]
*Terminalia arjuna* (Roxb.ex DC.) Wight & Arn. UBKV FOR 272	Asthma, heart problem, diabetes, stomach disorder, gastroenteritis, appetiser	Bark extract solution is administered orally to cure heart problem. Bark of *Terminalia arjuna*, *Alstonia scholaris* and fruits of *Terminalia chebula*, *Terminalia bellirica* and *Emblica officinalis* are powdered and its solution in a glass of water is consumed once a day early in the morning for 5–6 days as appetiser and cure gastroenteritis. Small pieces of bark is boiled in water and concentrated, cooled and taken in doses of two teaspoon of this cooled solution is consumed twice a day before meal or the bark pieces are powdered adding sweet candy and two teaspoon of this powder is consumed mixed in a glass of water to cure diabetes. Bark is soaked in water overnight and the solution is taken early in the morning for curing gastroenteritis and also as liver tonic. Half glass bark extract in water solution is administered orally twice a day for 5–7 days for curing breathing and heart problem.	Skin disease, leucoderma, liver disorder, fractured bones, tuberculosis**,** cardiac problem, hypertension, pimples and other minor skin eruption, cardio tonic [25, 41, 63, 86, 88]
*Terminalia bellerica* (Gaertn.) Roxb. UBKV FOR 45	Cough and cold, stomach disorder, indigestion, gastroenteritis	*Terminalia bellirica*, *Piper nigrum*, *Terminalia chebula,* cloves and *Cinnamomom camphora* are crushed together and made into tablets which are administered in empty stomach for curing cough. A glass of root extract is administered once a day for a week in empty stomach to cure stomach disorder. Besides the root xtract is also used to massage on stomach where pain is felt. Fruits are crushed into tablets and administered in empty stomach once a day to cure cough and cold. Fruit pulp extract is boiled (1 kg fruit in 2 l) and the hot solution is consumed for curing gasteroenteritis. Fruit pulp is crushed to tablets and taken empty stomach twice a day for a month for curing severe cough and cold. Dry fruit is crushed to powder and swallowed to cure stomach problem.	Skin disease, cold, constipation, leucoderma, grey hair, rheumatism, diarrhoea, dysentery, indigestion, dyspepsia, cooling agent, health tonic, cuts and wounds, tonsillitis, bronchitis, piles, dropsy, leprosy, headache, asthma [10, 11, 41, 48, 55, 62, 63, 73, 74, 78, 86]
*Terminalia chebula* Retz. UBKV FOR 46	Almost for all sickness, appetiser, cough and cold, gastroenteritis, jaundice, liver, pneumonia	Fruits of *Terminalia chebula*, *Terminalia bellirica*, *and Emblica officinalis* are dried and one fruit of each is soaked in water for 24 h and taken in empty stomach for making the liver strong and also as appetiser. Parts of plants are beaten to powder or is boiled and consumed twice a day for curing any kind of sickness. Fruits are crushed and mixed with water consumed for treatment of cold and cough. Two teaspoons dried fruit pulp powder is consumed twice a day in empty stomach for indigestion, cough and cold. Fruit pulp is boiled, cooled and the solution is consumed emptyty stomach (one glass a day for 4–5 days) for curing cough. Flowers are cooked and consumed to cure cough. Fruit pulp is boiled with black salt, crushed into tablets, sundried for few days and then consumed early in the morning to cure gastroenteritis. Fruits are soaked over night in water overnight and then the solution is taken in empty stomach as appetiser, cure jaundice and making liver strong. Fruits are also chewed to get relief from cough and cold as well as pneumonia.	Carminative, laxative, digestive, appetiser, constipation, piles, stomach disorder/pain, tonsillitis, diabetes, intestinal ulcer, rheumatism, urinary problem, skin problem, cold and cough, respiratory troubles, fever [10, 11, 25, 41, 42, 55, 62, 63, 73, 74, 78, 80, 86]
*Cuscuta europaea* L. UBKV FOR 311	Jaundice	The whole plant is boiled and either taken bath with light warm water once a day for 2–3 times or a glass of this solution mixed with salt is consumed as a therapy for jaundice.	–
*Ipomoea batatas* (L.) Lam. UBKV FOR 326	Tumours of the mouth and throat, asthma, burns, fever, diabetes	Rhizomes are cooked and consumed by diabetic patients	Diabetes, scorpion sting [12, 72, 76]
*Ipomoea carnea* Jacq. UBKV FOR 327	Fungal infection between toes and fingers	–	Fungal infection between toes [76]
*Bryophyllum pinnatum* Kurz. UBKV FOR 295	Burn injuries, cuts and wound	Leaves are grinded, applied on affected part and dressed.	Gall bladder stone, piles, stomach problems, antiseptic, kidney stone, skin disease [25, 88]
*Coccinia coridifolia* Cogn. UBKV FOR 307	Lowers blood pressure	Fruits are cooked as vegetables and taken with meal to lower blood pressure.	–
*Coccinia indica* L. UBKV FOR 308	Lowers blood pressure, gastroenteritis	Fruits are chewed or cooked as vegetables to lower blood pressure. Leaves are pasted and added with water. It is consumed for curing gasteroenteritis.	Hypertension, healing wound, ulcer, jaundice [26]
*Lagenaria siceraria* (Molina) Standl. UBKV FOR 330	Boils	Tender leaf extract are used to treat boils.	Jaundice, diarrhoea and dysentery [72, 75]
*Luffa aegyptiaca* Mill. UBKV FOR 332	Anaemia, liver disorder, menstrual problems	–	–
*Momordica dioica* Roxb. ex Willd. UBKV FOR 335	Stomach disorder, fever	Fruits are consumed to cure stomach disorder. One glass leaf extract twice a day is consumed for 2 days to cure fever.	Urinary trouble, piles, diabetes, jaundice, ulcer, dysentery, ear pain, breast swelling, hair vitalizer [55, 72–74, 83, 85]
*Dillenia indica* L. UBKV FOR 62	Stomach disorder	Bark is boiled and taken for curing stomach disorder.	Bark to get relief from indigestion, gasteroenteritis, diarrhoea [10, 55, 65, 73, 74]
*Dioscorea belophylla* Voigt UBKV FOR 65	Appetiser, jaundice, body pain, stomach disorder, pneumonia	Fruits are cut into pieces and soaked in water overnight, boiled and eaten as appetiser. Tuber is crushed and messaged on the whole body once a day to get relief from pain. Tuber is cut into pieces and partially boiled and is dip in water for a night. In morning it is fried as curry with little oil and eaten with meal for 3–4 days for curing pneumonia.	–
*Dioscorea bulbifera* L. UBKV FOR 312	Jaundice	Tubers are crushed and soaked in water for 5–10 min. One glass of this solution is consumed to treat jaundice.	
*Shorea robusta* Gaerth f. UBKV FOR 68	Cuts and wounds, burns, stomach pain, blood coagulant, dysentery	Bark powder/extract is and mixed with water to form solution and taken 3 times a day in empty stomach for curing stomach problems. Bark powder is applied on burns once a day for 3–4 days for relief.	Gonorrhoea, diarrhoea, dysentery, burning sensation, chest pain, pox, ear pain [25, 41, 55, 73, 74, 78, 82]
*Elaeocarpus ganitrus* Roxb. ex G. Don UBKV FOR 313	Piles, good sleep, asthma, cough	–	–
*Baccaurea remiflora* Lour. UBKV FOR 289	Skin disease	–	
*Baccaurea sapinda* (Roxb.) Müll.-Arg UBKV FOR 290	Tooth ache	Twigs are used to brush teeth to get relieve from tooth pain.	–
*Codiaeum variegatum* (L.) A. Juss. UBKV FOR 310	Gastroenteritis, ulcers, fencing, decorative, religious, fuel wood	The whole plant is simply used for fencing purpose, decorative purpose, after the plant is dried it is used as fuel wood, and the leaves is used as religious purpose (puja).	
*Emblica officinalis* Gaerth. UBKV FOR 263	Hair loss, liver problem, stomach pain	Raw fruits are eaten to get relief from stomach pain. Fruits of *Emblica officinalis*, *Terminalia chebula* and *Terminalia bellirica* are dried, crushed and made into powder. One teaspoon of this powdered in water solution is consumed daily early in the morning to restrict hair loss and to cure liver problem.	Constipation, fever, itching, digestive, gasteroenteritis, haemorrhage, jaundice [25, 64]
*Hevea brasiliensis* (Willd. ex A. Juss.) Müll.-Arg. UBKV FOR 323	Ornamental (landscape)	–	
*Jatropha curcas* L. UBKV FOR 264	Diarrhoea, headache	Sugar candy and few drops of *Jatropha curcas* latex are mixed and consumed for treatment of diarrhoea. Seeds are pasted with water and applied on forehead for curing headache.	Cuts and wounds, skin disease, seeds digestive, twigs used in tooth and gum problem, rheumatic pain, night blindness [10, 25, 35, 61, 75]
*Mallotus tetracoccus* (Roxb.) Kurz. UBKV FOR 333	Skin diseases, ring worm	–	–
*Ricinus communis* L. UBKV FOR 84	Cough, headache, dental problem, jaundice	Leaf extract is massaged in neck for 3 times a day for 5 days for curing cough; however during the therapy period non-vegeterian food is avoided. Seed oil extract is applied on head to get relief from headache. Leaf extract is mixed with sugar and consumed for 3–4 days to cure jaundice. Alternately, fruits are also boiled with grains of *Cajanus cajan* and consumed to cure jaundice. Twigs are used for brushing teeth for healthy dentals.	Piles, wounds (human and animal), joint pain, jaundice, headache, fever, boils, dysentery, stomach problems, indigestion, skin disease, hair loss, sores, boils, burns, rheumatic swelling, stomach worms [10, 11, 25, 55, 67, 68, 73, 74]
*Acacia catechu* (L. f.) Willd. UBKV FOR 251	Cuts and wounds, burns, sore throat, diarrhoea, digestion	Soft wood is cut in small pieces and boiled with water for some time and left for cooling to soilidify. The preparation is smeared with betel leaf for chewing to cure gastric problem.	Leaf for indigestion, bark, gum and root to control rheumatism [10]; body ache, fracture [55]; toothache, cough and cold, stomach pain [25]; astringent, boils and skin eruptions, diarrhoea and dysentery, bleeding from nose, chest pain [64–67]
*Acacia nilotica* (L.) Delile UBKV FOR 275	Evil spirit	Dental problems	Jaundice, itching, skin disease, worms, maggot wounds, dental problems, easy delivery, burns, asthma, fever, headache, stomach problems, indigestion, cholera, diarrhoea, dysentery [25, 63, 66, 67]; indigestion and gas problem of cattle [68]
*Bauhinia malabarica* Roxb. UBKV FOR 293	Stomach pain	Leaves are boiled and consumed daily for 2–3 months to get relief from stomach pain after pregnancy.	–
*Cajanus cajan* (L.) Mill UBKV FOR 296	Gall bladder stone, diabetes, jaundice and liver problem	Leaf extract is taken to cure diabetes. Fruits are eaten to get rid of gall bladder stone. Leaf extract solution is made with 200 ml of water and 100 g of either palm candy or sugar candy and administered orally once in the morning for 3 days in empty stomach. Roots are also used for curing jaundice by grinding them with water, sieved and 200 ml solution administered orally early morning in empty stomach for 3 days.	Jaundice, dysentery, ear pain [41, 83, 89]
*Cassia alata* L. UBKV FOR 299	Bees sting, allergy, appetiser for children, weakness	Leaves are crushed and applied on bee sting and infected area of skin infection. Bark extract is consumed for appetiser and health tonic.	Skin disease, body pain [35, 41]
*Cassia sophera* L. UBKV FOR 300	Dysentery, cuts and wounds, cough, blood purifier	Leaf, flower and root extract is consumed to cure dysentery, wounds, cough and purify blood.	Diabetes [40]
*Hippocrepis emerus* (L.) Lassen UBKV FOR 325	Skin infection	Leaves are crushed and rubbed on infected skin for curing.	–
*Mimosa pudica* L. UBKV FOR 93	Reddening of eye	Leaf extract not more than a drop is applied for curing red eye.	Orchitis and depurative, infertility, dental pain, head ache, kidney/urine trouble, piles, sores, diarrhoea, dysentery, hydrocoel, jaundice, wounds and swelling, placenta prolepses [10, 25, 55, 61, 64, 71, 73, 74, 76]
*Pongamia pinnata* L. UBKV FOR 344	Swelling, tooth pain	Seed oil extract is massaged to reduce swelling. Tender branches are used as tooth brush to cure dental pain.	Fever [67]
*Tamarindus indica* L. UBKV FOR 353	Neck pain, tonsils and swelling	Fruits are soaked in cold water for 2–3 days, mixed intensively and seeds are removed. The solution is taken a glass a day for curing neck pain, tonsil and swelling. Leaves can also be eaten for curing neck pain and swelling for both human beings and animals.	Diarrhoea/dysentery (human and animals), indigestion, fever, piles, ulcer, nausea, vomiting, swelling, jaundice, gastropathy, wounds, scorpion bite, scabies, stomach pain, boils, cold, cough, skeletal flurosis [11, 26, 27, 67]
*Trigonella foenum-graecum* L. UBKV FOR 356	Diabetes, joint pain	Whole plant is cooked and consumed to cure diabetes and joint pain. Seeds are also taken in empty stomach with water to curb diabetes.	Alopecia, diabetes, stomach problems, easier pregnancy, diarrhoea of animals, increase lactation of cattles, joint pain of animals, [22, 61, 68]
*Quercus castanopsis* H. Lév. UBKV FOR 346	Neck pain	Flowers are eaten for removing fish or meat bones stuck in the neck.	–
*Ganoderma lucidum* (Curtis) P. Karst. UBKV FOR 322	Asthma, lungs problem, lowers cholesterol	The fruiting body is cooked as vegetable and consumed to control the diseases/ailments.	–
*Clerodendrum viscosum* Vent. UBKV FOR 246	Skin disease, stomach worm, stomach pain, tooth pain	Leaves are boiled taken bath to get cure from body itching. Tender leaves and *Centella asiatica* are crushed, and the extract (1–2 teaspoons for children and adult, respectively) is consumed in empty stomach twice a day for 3 days to get relief from stomach worm and stomach pain. Twigs are used to brush the teeth to get relief from tooth ache.	Pain, skin disease [41]
*Gmelina arborea* Roxb. UBKV FOR 247	Appetiser, piles, abdominal pain, burning sensation, fever, headache, ulcer	Flowers are cooked and consumed to control the diseases/ailments.	Vomiting, diarrhoea, weakness, snake bite, cut and wound, piles [25, 41, 42]
*Leucas aspera* (Willd.) Spreng. UBKV FOR 331	Stomach disorder, swelling, headache, stop bleeding, headache, body pain, tooth pain, cuts and wounds	Half a cup leaf extract with salt is takenr 2–3 times a day to cure stomach disorder or swelling. Leaf extract or any plant part is inhaled to get relief from headache. Leaf extract is applied on cuts to stop bleeding and heal as well. Tender plant is cooked and consumed to get relief from body pain and gastroenteritis. Leaves are dried, mixed with raw rice and powdered, and half cup of its water solution is consumed in empty stomach daily for 2–3 days to remove swelling. Roots are chewed after dinner for 3–4 days to get relief from dental pain.	Cuts and worms of cattle [37]; skin disease, sore in thigh [90]
*Ocimum sanctum* L. UBKV FOR 267	Cough and cold, neck pain, cancer, gastroenteritis	Leaf extract of *Ocimum sanctum* and *Justicia adhatoda* are mixed with water, sieved and the solution is mixed with 5 ml honey which is consumed 3–4 times a day for 2 days to cure cough. Leaves mixed with ginger are chewed to get relief from cough and cold. Taking three leaves daily anytime with water in a day helps to keep cancer away. For curing gastroenteritis and cough, leaf extract can be taken any time in a day. Dried young stem are laced and tied around neck to avoid any disease.	Asthma, cold and cough, fever, bronchitis, genito-urinary disorders, diaphoteric, antiperiodic, stimulating [10, 11, 41, 64, 69, 82]
*Cinnamomom camphora* (L.) J. Presl UBKV FOR 303	Stomach disorder, diabetes, reduce weight	Dried bark or leaves can be taken as such for curing stomach disorder, diabetes and also helps in reducing weight.	–
*Machilus villosa* (Roxb.) Hook. f. UBKV FOR 361	Joint pain	Bark extract is placed on joints for an hour to get relief from pain.	
*Careya arborea* Roxb. UBKV FOR 125	Body pain, dysentery	Bark extract is sieved and solution is taken in empty stomach once a day for 3–4 days to cure dysentery. Bark extract is also administered externally to get relief from body pain.	Dysentery, cough, mouth and throat infection [41, 84]
*Lagerstroemia speciosa* (L.) Pers. UBKV FOR 138	Ulcers and sore, diabetes, piles, to obtain white and strong teeth,	–	
*Punica granatum* L. UBKV FOR 345	Nose bleeding	Leaves of *Punica granatum* and black cumin are grinded adding water and 2–3 drops are dropped in nose to stop nose bleeding. Fruits when ripe are consumed to increase blood.	Diarrhoea, dysentery, bronchitis, fever, indigestion, heart problem, eye and ear infection, jaundice, nasal congestion [25, 55, 64, 66, 73, 74]
*Abroma augusta* (L.) L.f. UBKV FOR 274	Night wetting, jaundice and stomach disorders	For night wetting—bark of *A. augusta* and early roots of *Bombax ceiba* are mixed, washed and grinded into paste with palm candy. The paste is mixed with required amount of water and sieved. The solution is taken in empty stomach twice a day till the problem persists. For jaundice—bark of roots is peeled and paste is made after grinding with sugar candy. The paste is mixed with water and stored overnight. The solution is taken in empty stomach during early hours of morning for 3 days. For stomach disorder—roots and barks are washed, grinded lightly and soaked in water for overnight to form a jelly-like solution. The jelly is taken in empty stomach once a day for 5 days.	Menstrual disorder, snake bite [55]; blood dysentery, diarrhoea, night wetting, [61]
*Hibiscus rosa sinensis* L. UBKV FOR 324	Tonsillitis, dandruff, hair loss	–	Mennorrhagia dysentery, fever, headache, burn, boil, skin disease, cough and cold, fatigue, hair fall, abortion, burning sensation, twitching [25, 41, 62, 72, 82]
*Melastoma malabathricum* L. UBKV FOR 144	Cuts and wounds, stomach ulcers, dysentery, diarrhoea, dental pain	–	Pneumonia, diarrhoea [55, 73, 74]
*Azadirachta indica* A. Juss. UBKV FOR 256	Allergy, fever, pneumonia, small pox, appetite problem, brushing teeth, stomach disorder, skin disease, tooth pain	Leaf extract in water solution taken twice a day in empty stomach to get relief from fever. Domestic animals is also treated same way for fever. Half a glass juice extract is also administered orally daily for 3–4 days to cure pneumonia. Leaf extract is also consumed as appetiser. Leaves are boiled in water and used for taking bath for curing itching, fever and pox. For curing fever, leaves of neem and rice are grinded to powder and taken 1 spoon twice a day in empty stomach. Leaves are fried and consumed to cure mouth ulcers. Twigs are used to brush the teeth to get relief from tooth pain and dental problems. Leaves are beaten into tablets, sun dried and taken in empty stomach for curing gastric, appetite problems, fever, and other stomach disorder.	Allergy, skin disease, fever, boils, cut and wounds, cough and cold, eye and ear infection, dental problems/tooth brush, leprosy, intestinal worms, ulcer, stomach, ear and tooth ache, acidity, vomiting, blood sugar, diabetes, malaria, blood purifier, heart problems, typhoid, health tonic, cancer [10, 22, 25, 27, 41, 55, 63, 72–75, 80–82, 86, 87]
*Melia azedarach* L. UBKV FOR 334	Fatigueness, cough, fever, appetiser, deworming, cuts and wound, vomiting, skin disease, dental problem, piles, pox, mosquito repellent, purify blood,	–	Blood purifier, reduce blood pressure, skin disease, head ache, fever, stomatitis, stomach worm, stone in urinary bladder, fever, antiseptic [12, 25, 68, 74, 79, 68]
*Tinospora cordifolia* (Willd.) Miers ex Hook. f. & Thom. UBKV FOR 146	Stomach pain, diabetes	Small pieces of roots are soaked in water overnight and the solution is consumed empty stomach early in the morning 1–2 times a day for 3–6 months to cure diabetes and stomach pain.	Rheumatism, jaundice, diabetes, burning sensation during urination, piles, eye infection, fever, diarrhoea, dysentery, malaria, skin disease, appetiser [10, 25, 55, 62, 72–74, 80]
*Entada rheedii* Spreng. UBKV FOR 315	Diarrhoea of domestic animals	The fruit pulp is crushed mixing with water and salt to feed domestic animals on banana leaf for treating diarrhoea.	Cuts and wounds, skin disease [55, 73, 74]
*Artocarpus hetrophyllus* Lam UBKV FOR 254	Skin diseases, asthma, ulcers	Mature leaves are chewed mixed with salt.	Antiseptic [26]; fever, boil, cut and wound, skin diseases, diarrhoea, toothache, snake bite [35, 55, 61, 64, 73, 74]
*Artocarpus lakoocha* Roxb. UBKV FOR 154	Skin ailments, headache	Paste of leaf and bark are applied on infected part	Leaf and bark to treat skin disease [10]
*Ficus benghalensis* L. UBKV FOR 316	Chronic diarrhoea and dysentery, piles	Latex in water solution is used orally for treatment of piles. Bark extract for diarrhoea/dysentery.	Diabetes, gout, dysentery, diarrhoea, asthma, muscular pain, gasteroenteritis, hair vitaliser, joint pain, sexual disorder, stomach pain, dental problem [12, 25, 26, 55, 63, 71, 73, 74, 86]
*Ficus carica* L. UBKV FOR 317	Swellings, tumours, ulcers	–	Constipation [20]
*Ficus elmeri* Merr. UBKV FOR 318	Fever	Leaves are boiled, and luke warm solution is taken for 1–2 days or leaf can be chewed for curing fever.	–
*Ficus geniculata* Kurz UBKV FOR 319	Diarrhoea	Tender leaves either after cooking consumed or dried, powdered and consumed in water solution to cure diarrhoea.	–
*Ficus hispida* L. f. UBKV FOR 320	Ulcer, jaundice, fever, liver problems	–	Control blood sugar level, mouth ulcer [35, 41]
*Ficus racemosa* L. UBKV FOR 321	Mouth disease of domestic animals	Leaves are fed to the cattle/goat to cure their mouth disease.	Leucorrhoea, piles, stomach pain, dysentery, fiver, ulcer [25]
*Morus alba* L. UBKV FOR 337	Jaundice, fever, appetiser for domestic animal	Roots are cut into small pieces, wrapped in cloth and tied on neck for 12 days or leaves are boiled and after cooling the solution is consumed once a day to cure jaundice.	Sore throat, cough, brain and heart tonic [55, 73, 74]
*Moringa oleifera* L. UBKV FOR 336	Blood pressure, gastroenteritis, cold and cough, body pain, clearing stool, cuts and wounds of domestic animals, snake repellent	Leaf extract is taken to cure high pressure and gastroenteritis (1 glass for 2–3 months daily). Root extract are helpful to cure cold and cough and heal wounds of domestic animals. Pods are eaten as vegetable which also help to regulate blood pressure and body pain. Tender leaves are also cooked as vegetables to cure gastric, body pain, digestion and clearing stool. Roots are cut into small pieces and spread around the house as snake repellent.	Tumours, leucoderma, liver disorder, snake bite, piles, cough, stomach worm, diarrhoea, dysentery [25, 55, 73, 74, 81]
*Musa balbisiana* Colla UBKV FOR 338	Liver problem, gastroenteritis, dysentery, swelling, body severing, stomach disorder, teeth pain	Dark black roots of banana and *Clerodendrum viscosum* are crushed to paste and placed on chicks facing sunlight for an hour to get relief from dental pain. A full teaspoon of latex is consumed three times a day for 3 days to cure loose motion. Fruits or flowers are cooked and taken with daily meal to curing stomach disorder. Roots of banana are used for a night to reduce swelling and control body shiveringt. Ripe fruits or a cup of pseudostem sap is orally taken once for curing dysentery. One cup tender leaf extract solution is taken empty stomach early in the morning for at least 15 days to cure liver problems and dysentery. Fruit peel is pasted, mixed with glass of water and is consumed once in a day in empty stomach for about 15 days for curing gastroenteritis.	
*Myristica longifolia* Wall. UBKV FOR 165	Stop bleeding, cough	Roots are soaked for few minutes and boiled in water till it turns red in colour. One cup of this solution after cooling is consumed as health tonic in empty stomach once a day for 3 days. The solution is also fed to domestic animals. Roots are crushed; boiled with water, cooled and one cup of this solution is taken daily twice for 3 days to stop menstrual bleeding. Root extract water solution of young plants is taken to cure cough.	–
*Psidium guajava* L. UBKV FOR 270	Dysentery/loose motion, stomach pain	Ripe fruits or tender leaves are eaten for curing dysentery. Tender leaves are chewed in empty stomach for 3–4 days or can be crushed and taken with adding water for curing stomach pain. Tender leaves extract (2–3 spoons) is taken for 2–3 times a day till loose motion is cured.	Cold and cough, fever (human and animal), indigestion, dysentery, diarrhoea (human and animal), ulcer, vomiting, dental pain, joint pain, dental pain [10, 11, 25, 41, 55, 64, 73, 74]
*Syzygium cumini* (L.) Skeels UBKV FOR 169	Cough and cold, blood dysentery, gastroenteritis	Fruits are consumed to cure cough and cold, blood dysentery and gastroenteritis.	Indigestion, dysentery, diarrhoea, loose motion, stomach pain, diabetes, anti-inflammatory, sore throat, bronchitis, ulcers, joint pain, piles [10, 11, 25–27, 62, 63, 65, 80, 82]
*Nyctanthes arbor-tristis* L. UBKV FOR 339	Fever	Leaf is either rubbed or its two teaspoon extract is consumed 2–3 days for curing fever. Flowers are also used as medicine following the same procedure.	Rheumatism, malaria, fever, cough and cold, fracture, [25, 88]
*Averrohoa carambola* L. UBKV FOR 288	Jaundice and liver problems	A glass of pulp juice is consumed three times a day in empty stomach for week to get a relief from jaundice.	–
*Piper nigrum* L. UBKV FOR 268	Cough and cold	Dried fruit powder in water solution is consumed for curing cough and cold.	Asthma, cold and cough, rheumatism, constipation, eye infection, throat infection, indigestion, piles [10, 41, 42, 55, 61, 64, 73, 74, 83]
*Plumbago zeylanica* L. UBKV FOR 342	Hydrocoel, fever	Roots are tied with arms or thigh for 20–30 min during morning to cure hydrosol. Rhizomes are crushed, boiled and half a glass taken early in the morning daily for 5–6 days to treat fever.	Eroenteritis, skin diseases, scabies, ulcer, diarrhoea, dysentery, indigestion, night blindness, abortion, leprosy [55, 62, 64, 73, 74, 84]
*Arundo donax* L. UBKV FOR 287	Headache		Skin disease [19]
*Bambusa vulgaris* Schrad. UBKV FOR 291	Night wetting	The nodes of bamboo after cutting are left in the open for 2 days. The water in the nodes is collected after 2 days and given to young children for night wetting.	
*Cynodon dactylon* (L.) Pers. UBKV FOR 261	Cuts and wounds, bleeding, vomiting, nose bleeding, increase lactation of domestic animals	The grass is crushed, and extracts are applied on wounds to stop bleeding and healing as well. *Cynodon dactylon* and leaves of *Ocimum sanctum* are crushed together, and two teaspoons of this extract is taken orally to stop vomiting. Water solution of this extract is made mixing palm candy and half a cup of this solution is consumed once a day to stop nose bleeding.	Shoot and leaf juice to control vomiting, skin disease, leprosy, piles, asthma, arthritis, burning urination, indigestion, cancer, eye and mouth problem, dysentery, blood purifier, control nose bleeding, anti-septic, snake bite, stop bleeding, wounds, miscarriage [10, 12, 20, 25, 55, 66, 73, 74, 84]
*Eleusine indica* (L.) Gaertn. UBKV FOR 314	Weakness of children	Whole plant is crushed with water, and 2–3 teaspoons of this solution is administered orally once a day for 2–3 days as tonic for general weakness.	
*Thysanolaena latifolia* (Roxb. ex Hornem.) Honda UBKV FOR 355	Tonsillitis, boils, abortion	–	Boils, burns, tonsillitis, abortion [12]
*Triticum aestivum* L. UBKV FOR 357	Hair loss	Half a glass tender leaf extract are consumed daily early in the morning for a month to prevent hair fall.	Flatulence in goats [68]
*Polygonum dichotomum* Blume. UBKV FOR 269	Baby crying	Tender leaves of *Opuntia ficus-indica* and *Polygonum dichotomum* are mixed with milk and crushed together. Few drops are given for 2 times a day to stop baby crying.	Leaves and tender shoots against dental problem [10]
*Polygonum hydropiper* L. UBKV FOR 343	Dysentery	–	Blood coagulant, dysentery, gasteroenteritis, astringent [92, 93]
*Anthocephalus cadamba* (Roxb.) Miq. UBKV FOR 285	Asthma	Leaves of *Anthocephalus cadamba* along with alum powder are boiled together and then the water after is taken half cup 2 times a day for 2 days and again after 1 month taken for 2 days in the same quantity of dose (i.e. should be continued in interval of every months which helps in curing asthma.	Cholera, diarrhoea, dysentery, pyorrhoea, tooth brush, tooth pain, pimples, sores [11, 55, 73, 74]
*Aegle marmelos* (L.) Corrêa UBKV FOR 218	Stomach disorder, appetiser, dysentery	One glass of fruit pulp juice is taken three times a day to cure dysentery, diarrhoea and other stomach problems. It is also used as appetiser.	Fever, piles, diarrhoea and dysentery, digestion, head ache, stomach ache, gastroenteritis, cough, asthma, tumours, mouth ulcer, [23, 25, 26, 63, 72]
*Citrus limon* (L.) Osbeck UBKV FOR 306	Vomiting, dysentery, blackening of nerve of children after birth	Leaf or root extract of C. lemon, bark of papaya and roots of *Aegle marmelos* are administered externally once a day for 3–4 days for curing blackening of nerve of children. Leaves and fruits are either smelled or consumed as raw to avoid vomiting.	Stomach ache, dysentery/diarrhoea, stomach worms, dandruff [64, 72, 76]
*Murraya koenigii* (L.) Sprengel UBKV FOR 266	Gastroenteritis	Leaaves are consumed raw or as extract once a day in empty stomach to cure gasteroenteritis.	Leaf extract for black fever and diarrhoea, dysentery, diabetes, anaemia, vomiting, cut and wound, inflammation, appetiser [10, 25, 26, 61, 72]
*Scoparia dulcis* L. UBKV FOR 347	Piles, paralysis, diarrhoea, gastroenteritis	Leaf, stem, root or flower extract are consumed (2 teaspoons/day for 3 months) to cure piles. These parts are powdered and applied in the nose (2 times a day for 3 months) for treatment of paralysis. Plant (without root) extract solution is consumed in empty stomach (1 glass daily for 5 days) for curing diarrhoea and gasteroenteritis.	Cough, burning sensation in pulmonary artery and veins, painful urination, diabetes, bronchitis, piles, cough, fever, tumour, boils, pneumonia, kidney stones, antiseptic, menstruation disorder [41, 55, 64, 73, 74, 83, 86, 87]
*Ailanthus integrifolia* Lam. UBKV FOR 224	Fever	Resin extract from the plant is lightly burnt for fumigation of patient to get relieve from fever.	
*Datura metel* L. UBKV FOR 262	Diarrhoea, dental pain, ear pain, cut and wounds, fatigueness/weakness of domestic animals	Fruits are roasted and consumed to cure diarrhoea. Root extract is mixed with water to feed domestic animals as general health tonic. The fruit pulp is mixed with coconut oil and heated which is then applied on ear after cooling twice a day for 2 days to get relief from pain. Unripe fruits are used to heal wound. Fruits or seeds crushed with mustard oil and heated and applied on teeth during morning for 2–3 days to get relief from pain.	Asthma, boils, leprosy, diarrhoea, piles, cold and cough, joint pain, antirabies, gout, sores, dandruff, hair loss [10, 55, 61, 64, 73, 74, 76]
*Solanum indicum* L. UBKV FOR 348	Pneumonia, diabetes	Root beaten and made in the form of tablets to cure pneumonia. Fruits are cooked and eaten as vegetables for curing diabetes.	Ringworm in cattle [70]
*Solanum khasianum* C.B. Clarke UBKV FOR 230	Dental pain	Fruits are burnt and placed on the teeth for getting relief from pain.	Dental pain [41]
*Solanum melongena* L. UBKV FOR 349	Antiseptic	Fruits are cut and rubbed for a week to treat infection.	–
*Solanum nigrum* L. UBKV FOR 350	Dysentery, vomiting, asthma, bronchitis, fever, urinary discharge, cuts and wound	–	Wound, jaundice, abdominal swellings, stomach pain, head ache, fever, gonorrhoea, piles, dysentery, boils, eye infection [27, 35, 42, 55, 73–76]
*Solanum xanthocarpum* Schrad. & J.C. Wendl. UBKV FOR 362	Stomach disorder, blood purifier, eye problem	Bark extract is boiled in water or fruits are eaten after ripening for purifying blood and cure gastroenteritis and dysentery. Fruit juice is applied twice a day to control eye problem.	–
*Sterculia villosa* Roxb. UBKV FOR 233	Dysentery	–	Seminal weakness [84]
*Christella dentata* (Forssk.) Brownsey & Jermy UBKV FOR 258	Cuts and wound	Leaves of *Christella dentata*, *Justicia adhatoda* and *Clerodendrum viscosum* are boiled and the solution is used to wash wounds till healed. The plant is also used as housefly and insects repellent.	Cuts and wounds [10]
*Typha elephantina* Roxb. UBKV FOR 358	Urine problem, purifies breast milk, internal bleeding	–	–
*Lantana camara* L. UBKV FOR 250	Cough and cold	Leaf extract is taken orally (1–3 spoon 3 times a day) or the leaves are chewed or consumed with rice to cure cough and cold.	
*Tectona grandis* L. f. UBKV FOR 248	Dysentery, piles, diabetes, prevent unwanted hair growth, construction, furniture, agricultural implements, fodder	Young leaves are used as fodder.	Flowers used in bronchitis and urinary discharges; to promote hair growth along with coconut oil; leaf sap used to treat irregular menstrual cycle, burning sensation, kidney and skin disease, headache, tooth ache, swelling, stomach burning, skin irritation [25, 41, 63, 82, 86]
*Vitex negundo* L. UBKV FOR 273	Fever	Half a glass leaf extract solution in water is taken twice a day (before meal in morning and after meal at night) for 3 days to treat fever before meal in the morning and after meal at night.	Gout, fever, headache, cough and cold, sinusitis, diarrhoea, cardiac problems, rheumatism, fat burning, bedsore, wound, backbone pain, fracture, body swelling, joint swelling greying hair, memory vitaliser, stomach problem, swelling of joints, liver problem, jaundice [10, 12, 41, 42, 48, 55, 61, 63, 64, 66, 73, 74, 77, 78, 86, 91]
*Cissus quadrangularis* L. UBKV FOR 304	Fracture, paralysis, leg pain	Whole plant is crushed and dressed on the fractured body part for 24 h. The process is repeated for three times or till recovery. The therapy is same for domestic animals also.	Stem extract is used to treat broken bone [25, 41, 67, 82]
*Cissus repanda* Vahl UBKV FOR 305	Bone fractures, cuts and wounds	–	–
*Aloe vera* L. Burm.f. UBKV FOR 279	Fire burnt skin, stomach disorder, body pain	Pulp is used for curing burnt skin. The entire plant can be grinded for juice extraction and consumed to get relief from stomach disorder. Half a glass extract is also consumed daily early in the morning adding water and salt for relief from body pain.	Diabetes, general health, cough and cold, burns, cut and wound, fracture, arthritis, backbone pain, hepatitis, dermatitis [22, 25, 66]; stomach ache, piles, intestinal worms, eye disease, skin disease, allergy, tumours, liver tonic, purgative [69]
*Alpinia malaccensis* (Burm.f.) Roscoe UBKV FOR 280	Cuts and wounds, sores, digestive	Inner part of the leaf is eaten as vegetables to help digestion.	–
*Curcuma caesia* Roxb. UBKV FOR 259	Period of woman, chest pain, stomach pain, gastroenteritis, cuts and wounds	Fruits are meshed with salt and consumed bu. woman to relief from periodic pain. It is taken in empty stomach (1–1.5) teaspoon for 3 days. Tuber extract is mixed with water and taken to get relief from chest pain, stomach pain and gastric. The extract is applied on wounded part and dressed for 2 days.	Dried rhizome powder to treat skin disease, bone fracture, rhizome in rheumatic pains [10, 41, 81]
*Curcuma longa* L. UBKV FOR 260	Lactation problem of domestic animals, crack on legs, cough and cold, fever, stomach disorder, cuts and wounds	Flowers are crushed, mixed with feed and fed to animals for increasing the milk production. Alum and lime is crushed to powder and mixed with unripe rhizomes and placed on ruptured portion of the skin for 20 min and then washed, dried and messaged with coconut oil is used for massage once a day for 3 days till recovery. Fresfh rhizomes are roasted in mustard oil for few minutes and applied on wounds. The process continues till the wound is healed. Alternately, the rhizomes are boiled and consumed for healing of wound and relief from fever, cough and cold. Fresh rhizome extract is added with water and a cup of the solution is consumed after dinner for to stomach disorders like gastric pain. This solution is also consumes as health tonic.	Dried and fresh rhizome powder to treat cuts and wound, skin disease, inflammation, cough and cold, sores, boils, bone fracture, swelling, muscular or body pains, snake bite, stomach ache, bronco asthma, antidote against poison, blood purifier, jaundice and liver disorder [10, 12, 41, 55, 64, 73, 74, 76]
*Zingiber zerumbet* (L.) Sm. UBKV FOR 359	Snake bite, septic, cuts and wounds, back pain, pregnancy	Rhizome is pasted and placed on any bites or affected parts. Also used as massage to remove body pain or back pain. Rhizomes with leaves of *Raulfia serpentina* and tubers of *bedodgumi* are crushed together. This extract is mixed with half cup of water and consumed twice a day after meal for 2 days which helps to boost pregnancy.	–

**Table 3:** Number of species used as ethnomedicine for a particular disease/ailment

Human diseases/ailments	Species used
Stomach-related disorder (stomach problem, dysentery, diarrhoea, indigestion, stomach pain)	40
Cuts and wounds	27
Pain (joints pain, body pain, chest pain, neck pain, tooth pain)	25
Jaundice/liver problem	19
Cough and cold	18
Gastroenteritis	17
Fever	16
Skin-related disease	13
Diabetes	12
Appetiser and ulcer	9 each
Headache, piles,asthma and bleeding (internal, nose)	8 each
Burns	7
Swelling and pneumonia	6 each
Hair loss/fall/dandruff problems, blood pressure and paralysis	5 each
Vomiting and bite (dog, bees, snake)	4 each
Urine disorder, blood purifier and blood disease, sore throat and sores, tonsil	3 each
Small/chicken pox, malaria, hydrocoel, tumours, cancer, septic, boils, eye infection/conjunctivitis/red eye, clearing stool, reduce weight, tuberculosis, heart problems, night wetting, periodic problems of women	2 each
Typhoid, baby cry, dehydration, bronchitis, urinary discharge, abortion, gall bladder stone, pulmonary/lungs problem, anaemia, purifies breast milk, pregnancy, blackening of nerve, cholesterol, alcoholic medicine, fatigueness, body severing, body massager	1 each
Veterinary/disease/ailments	
Tongue/mouth disease and lactation problem	2 each
Stomach worm, cough, diarrhoea, cuts and wounds, fatigueness/weakness and appetiser	1 each

Among the documented species, 92 species were used to cure multiple problems, while the rest were used to cure single disease each (Tables 2 and 3). Similar observation was also reported by [40]. The fungus *Ganoderma lucidum* is used for asthma and lung problem. It also lowers cholesterol. According to the respondents, *Terminalia chebula* is used to treat almost all diseases and mainly is used as an appetiser and to cure gastroenteritis, jaundice, liver, pneumonia, cough and cold. The maximum number of 12 diseases/ailments was cured by *Melia azedarach* followed by *Centella asiatica* and *Rauvolfia serpentina* which were used to cure 11 diseases/ailments each.

The majority of the plant species (108) had more than one part that was medicinally important (Table 2) as was also documented by [41]. The indigenous communities mainly used the leaf of the plant for their ethnomedicinal uses as this part was maximum used with 83 species followed by the fruit (55 species), and least was the seed with four species (Fig. 1a). The leaves of the ethnomedicinal plants were also documented to be used by the majority of remedies in traditional medicines in several reports [20, 41]. The fruit was also reported as dominant and widely used part for traditional medicines [11, 26]. The other parts used were branch (32 species), stem (28 species), bark (26 species), flower (23 species) and rhizome/tuber (eight species). The whole plant of 24 species was used for ethnomedicinal purposes. Destructive harvesting is done when the whole plant is used. Even the sap, latex, resin and pulp of the plant species were also used. Harvesting patterns of the leaves or foliage, root, rhizomes and tubers indicate their possibility of vulnerability for becoming endangered as was earlier observed [10].

Proper selection of species, parts, as well as preparation and administration methods were very important in traditional healthcare systems. Ethnomedicinal formulations were administered both externally (skin, nasal, eye and dental) and internally as oral doses (Table 2) as was also observed by [10]. Most of the preparations were a mixture of different plant species, and in few cases, only one plant species was used. Different parts of a single species were also used to cure different diseases. Almost all plant parts were used to prepare different medicinal formulations: roots, rhizomes, tubers, bark, leaves, flowers, fruit, seeds, young shoots, whole plants, and gum and latex. Doses of these preparations were not standardised but administered on the basis of age, physical appearance and intensity of the illness. Children were usually administered with smaller doses than adult. The course of frequency of treatment is decided by the type of disease and its severity.

Mode of preparation included juice, paste, decoction, powder, infusion and chewing raw plant parts. The administration of the therapy is raw, dried form in small pieces or powdered, solution or mixed with water/milk/honey and paste/lotion. Generally, fresh part of the plant is used for the preparation of medicine [42]. The majority of formulations were prepared as juice followed by paste and decoction. Usually, the underground parts were used in dried form as was also earlier reported [40, 43]. The preference for roots and rhizomes to prepare traditional remedies follows the scientific basis that roots generally contain high concentrations of bioactive compounds [44]. There are several reports on the administration of ethnomedicine by various authors [11, 12, 20, 22, 41]. It was also observed that herbal treatment is still preferred by the residents for bone fracture and dislocation over modern treatment. Senior citizens trust more upon traditional treatment system over the modern methods as they believe no side effect with the traditional ethnomedicine. Similar observations were also documented by [10]. The present study documented 140 ethnomedicinal plant species from North Bengal, of which 62 species were also reported in earlier studies [19, 20, 39, 45–49] from north India with similar ethnomedicinal uses. The medicinal uses of the species also reported from north India is compared with our study and is presented in Table 4.

**Table 4:** Medicinal uses of the species in present study versus north Indian studies

Scientific name	North Indian studies
*Abroma augusta* (L.) L.f.	Urinary infection, bronchitis, diabetes [45]
*Areca catechu* L.	Boils and skin eruptions, diarrhoea and dysentery, bleeding from nose, chest pain [45, 46]
*Acacia nilotica* (L.) Delile	Jaundice, burns, headache, cholera, diarrhoea, dysentery [39, 45]
*Acorus calamus* L.	Dysentery and diarrhoea [46]
*Aegle marmelos* (L.) Corrêa	Fever, diarrhoea, dysentery, gastroenteritis, sun burn [39, 45, 46]
*Ageratum conyzoides* L.	Muscular pain, cuts, wounds, stop bleeding, piles, snake bite [45, 48]
*Aloe vera* L. Burm.f.	Stomach ache, piles, intestinal worms, eye disease, skin disease, tumours [46]
*Alstonia scholaris* (L.) R. Br.	malaria, pneumonia, stomach a che [45]; bark to treat snake bite, asthma and cardiac troubles, latex is applied to ulcers, sores and tumours [46]
*Andrographis paniculata* (Burm.f.) Wall. ex Nees	Malaria, fever, stomach problems, worms, dysentery [45, 46]
*Annona squamosa* L.	Diarrhoea, dysentery, indigestion, dandruff, lice, spinal problem [45, 46]; Constipation and abdominal swelling of animals [47]
*Anthocephalus cadamba* (Roxb.) Miq.	Cholera, dysentery, pimples [45]
*Artocarpus hetrophyllus* Lam	Cough, leprosy, ulcers [46]
*Arundo donax* L.	Skin disease [19]
*Asparagus racemosus* Willd.	Bleeding nose, epilepsy, arthritis [39, 45, 49]; fever and constipation of domestic animals [47]
*Azadirachta indica* A. Juss	Wounds, cold, diabetes, cancer [39, 45]
*Basella alba* Stewart.	Constipation, nose sorosis [45]
*Bombax ceiba* L*.*	Tooth ache, pimples, snake bite [45, 46]
*Calotropis procera* R. Br.	Pox, cholera, malaria, boils, skin disease, pregnancy, digestive tonic, indigestion of domestic animals [39, 45–47]
*Carica papaya* L.	Dysentery, tooth ache [48]
*Centella asiatica* (L.) Urb.	Leprosy, cough ulcer [46]
*Cissus quadrangularis* L.	Stem extract is used to treat broken bone [46]
*Citrus limon* (L.) Osbeck	Fever and mouth disease of animals [46]
*Clerodendrum viscosum* Vent.	Tumour, malaria, snake bite, boils, burns, cut and wounds [45, 63]
*Colocasia esculenta* (L.) Schott	Stop bleeding, removes redness of skin [46]
*Curcuma longa* L.	Cuts and wound, bone fracture, swelling, muscular or body pains, snake bite, stomach ache, blood purifier [46, 49]
*Cynodon dactylon* (L.) Pers.	Dysentery, snake bite, stop bleeding, wounds, [45, 49]
*Datura metel* L.	Asthma, boils, leprosy, diarrhoea, piles [45, 47]; cold, pneumonia of cattle [39, 47]
*Ficus benghalensis* L.	Diabetes, hair vitalizer, stomach pain, [39, 45]; diarrhoea and dysentery of domestic animals [47]
*Ficus carica* L.	Boils, skin disease, constipation [20, 45]
*Ficus hispida* L. f.	Mouth ulcer [45]
*Ficus racemosa* L.	Piles, boils, diarrhoea, dysentery, [45, 46]
*Hibiscus rosa sinensis* L.	Fever, abortion, burning sensation, twitching [39, 46, 49]
*Jatropha curcas* L.	Branches used as tooth brush to remove tooth pain [63]
*Justicia adhatoda* L.	Cough, bronchitis, asthma, malaria [45, 63]
*Mangifera indica* L.	Dysentery, ear ache, vomiting, heat stroke, digestion [39, 46]
*Melia azedarach* L.	Skin disease, head ache, stomach worm [46]
*Mimosa pudica* L.	Dental pain, head ache, wounds placenta prolepses [45, 47]
*Momordica dioica* Roxb. ex Willd.	Diabetic, breast swelling [45]
*Moringa oleifera* L.	Diarrhoea, dysentery [39]; flowers to restore health and proper urination, fruits for paralysis and diseases of the liver [46]
*Morus alba* L.	Fruits good for kidney and liver [46]
*Murraya koenigii* (L.) Sprengel	Fever, diarrhoea, dysentery, appetiser [46]
*Nyctanthes arbor-tristis* L.	Fever, cough dysentery, leaves for indigestion [45, 46]
*Oroxylum indicum* (L.) Benth.	Cough, mouth sore, ulcer, bronchial asthma, joint pain, appetiser [46]
*Plumbago zeylanica* L.	Diarrhoea, indigestion [45]
*Pongamia pinnata* L.	Malaria, fever, piles, cough, skin disease and boils [46]
*Psidium guajava* L.	Wounds, fever, diarrhoea ulcer, vomiting, [39, 46, 47]
*Punica granatum* L.	Diarrhoea, indigestion [46]
*Rauvolfia serpentine* (L.) Benth ex Kurz.	Insanity, blood pressure, intestinal disorder, vomiting, snake bite [45, 46]
*Ricinus communis* L.	Wounds (human and animal), joint pain, sores, boils, burns, rheumatic swelling, [45–47, 70]
*Shorea robusta* Gaertn f.	Diarrhoea, dysentery, burning sensation, chest pain, pox, ear pain [45, 48]
*Solanum khasianum* C.B. Clarke	Dental pain and decay [46]
*Solanum nigrum* L.	Wound, jaundice, abdominal swellings, stomach pain, head ache, fever, gonorrhoea, piles, dysentery, boils, eye infection [20, 45, 47]
*Syzygium cumini*(L.) Skeels	Dysentery, sore throat, bronchitis, ulcers, joint pain [39, 46]
*Tabernaemontana divaricata* (L.) R. Br. ex Roem. & Schult.	Eye disease, appetiser, dental pain [46]
*Tagetes erecta* L.	Hydrophobia [39]
*Tectona grandis* L. f.	Bronchitis, urinary discharges, headache, swelling, stomach burning, [46]
*Terminalia arjuna* (Roxb.) Wight &Arn.	Dysentery, tumours, asthma, fractured bones, diabetes, anaemia, hypertension [46]
*Terminalia bellirica* (Gaertn.) Roxb.	Skin disease, cold, constipation, cuts and wounds, piles, dropsy, leprosy, headache, [45, 46, 48]
*Terminalia chebula* Retz.	Constipation, stomach disorder/pain, urinary problem, cold respiratory troubles [45, 48]
*Tinospora cordifolia* (Willd.) Miers ex Hook. f. & Thom.	Jaundice, fever, diarrhoea, dysentery, malaria, appetiser [45, 46]
*Trigonella foerum-graecum* L.	Easier pregnancy, foot and mouth disease of animals, diarrhoea of animals, appetiser, [39, 47]
*Vitex negundo* L.	Headache, rheumatism, body swelling, joint swelling, diarrhoea of cattle [39, 46]

### Use value

The use value of a species indicates the ethnobotanical importance of a particular species in an area or by a community. The higher the value for a species, the higher is the importance of the species i.e. were most utilised or exploited. The use value range found was 0.01–0.13 (Table 1) The highest use value of 0.13 was estimated for *Centella asiatica*, followed by *Terminalia arjuna* and *Oroxylum indicum* each with 0.09; *Musa* sp*.*, *Ocimum sanctum* and *Leucas aspera* each with 0.08; and *Dillenia indica* with use value of 0.07. Use values of 0.05 were found for *Justicia adhatoda*, *Mangifera indica*, *Diplazium esculentum*, *Dioscorea belophylla* and *Azadirachta indica*, while *Terminalia bellirica*, *Morus alba*, *Moringa oleifera*, *Cynodon dactylon* and *Aegle marmelos* had a use value of 0.06. Use value on medicinal ethnobotanical plants was also earlier reported and similar conclusions made [50–52]. These species were utilised because of their therapeutic uses in multiple diseases and were abundantly available in wild and were also all grown in the home gardens.

### Conservation status

Various authors have feared that these ethnomedicinal species are disappearing from the wild due to unsustainable exploitation of these species and destruction of habitat of these species due to deforestation. Workers had reported the conservation status of medicinal plants of the Terai region [53] and Darjeeling Himalayas [54, 55] of West Bengal. In this study, the documented plant species were compared with these reports for the conservation status of these plant species. It was found that 38 ethnomedicinal plant species documented in this study were reported of their conservation status from Darjeeling Himalayas also (Table 1). According to the Darjeeling studies, plant species were classified as abundant, common, endangered, frequent, planted, rare and sparse and the number of documented species falling in these categories were four, five, three, five, five, three and 13, respectively (Table 1). Another study from the Terai region of West Bengal [53] also classified the plant species in terms of conservation status like rare, frequent and sparse, and 31 ethnomedicinal plant species documented in this study (Table 1) were categorised according to their conservation status in the Terai study.

Many plants growing wild and traditionally used are endemic and have become rare, threatened or endangered [56, 57], so they need to be conserved. Reserves of ethnomedicinal plants in developing countries are diminishing and in danger of extinction as a result of growing trade demands for cheaper products and new plant-based therapeutic markets in preference to more expensive target-specific drugs and biopharmaceuticals [11]. Genetic biodiversity of ethnobotanic plants is continuously under the threat of extinction as a result of commercial exploitation, grazing, environment-unfriendly harvesting techniques, loss of growth habitats and unmonitored trade of medicinal plants [58–61] This is because ethnomedicinal plants were freely harvested by users from their immediate environment either for their own use or traded domestically [62, 63]. The harvesting of these multiple use species can put them under threat [62] but can also lead to better chances for their conservation [63] especially through home gardens.

## Conclusions

The Chilapatta Reserve Forest and its fringe areas are rich in biodiversity of ethnomedicinal plant species. A total of 140 plant species represented by 116 genera and 65 families were documented for medicinal purpose. Majority of the plant species (108) have more than one part that was medicinally important. The indigenous communities mainly used the leaf of the plant for their ethnomedicinal uses. The curing of 58 human diseases from these documented plant species itself explains the importance of this area in national and international interest. Gastric problem is common in this area and 40 plant species were used for the treatment of this disease. *Centella asiatica* and *Rauvolfia serpentina* were the most valuable species in terms of its maximal use with higher use value. Comparison with the previous regional ethnomedicinal studies, we observed that 30 plant species documented in the present study were not having earlier reports. This means that the use of 30 plant species have been reported for the first time from this area for ethnomedicinal use. It was found that 38 ethnomedicinal plant species documented in this study were earlier reported for their conservation status from adjoining areas including endangered, frequent, planted, rare and sparse. The communities should be encouraged with improved cultivation techniques of commercially viable ethnomedicinal species through capacity building, timely policy intervention along with strong market linkage. This will ensure income generation and livelihood improvement and ultimate conservation of these species. The present information may serve as a baseline data to initiate further research for newly reported species for new compounds and biological activities which can be of immense value for societies to survive.
